# Development of an open-access food atlas with nutrient composition and recipes in Argentina: The Smart24Food Atlas

**DOI:** 10.1371/journal.pone.0359331

**Published:** 2026-09-25

**Authors:** Ismael Alejandro Contreras-Guillén, Sara Leeson, Rocio Victoria Gili, Belén Carlino, Emanuel Irrazabal, Sandaly Oliveira da Silva Pacheco, Fabio Juliano Pacheco

**Affiliations:** 1 Interdisciplinary Center for Research in Health and Behavioral Sciences, School of Medicine (CIICSAC), Universidad Adventista del Plata, Libertador San Martín, Argentina; 2 Faculty of Engineering, Universidad Nacional de Entre Ríos, Oro Verde, Argentina; 3 National Scientific and Technical Research Council (CONICET), Buenos Aires, Argentina; 4 Faculty of Exact and Natural Sciences and Surveying, National University of NorthEast (UNNE), Corrientes, Argentina; Lusofona University of Humanities and Technologies: Universidade Lusofona de Humanidades e Tecnologias, PORTUGAL

## Abstract

**Introduction:**

Accurate dietary intake assessment remains challenging, particularly for portion-size estimation, and context-specific photographic aids remain limited in Argentina. This study aimed to develop an open-access digital atlas of foods and recipes consumed in Argentina, integrating portion-size photographs and nutrient-composition data, and to describe its performance for food and recipe retrieval and visual portion-size matching under controlled direct-observation conditions.

**Methods:**

The Smart24Food atlas was developed as a digital resource integrating food photographs, standardized recipes, and nutrient-composition information. A controlled direct-observation study in a university setting included 108 participants, mostly young adults, completing 20 tasks: seven packaged-product selection tasks and 13 visual portion-matching tasks. Exact matching was analyzed using cross-classified logistic mixed-effects models, while weight-based performance was assessed using linear mixed-effects models and repeated-measures Bland–Altman analysis.

**Results:**

The atlas contains 3,273 photographs representing 998 distinct foods through 1,052 presentation-specific items, with nutrient-composition information for up to 53 components. Food or recipe retrieval was successful in 99.81% of tasks (2,156/2,160), and overall exact matching was 86.5% (1,869/2,160). Food-level heterogeneity was greater than participant-level heterogeneity, whereas food category and task type were not significantly associated with exact matching. Across 1,404 visual portion-matching observations, mean signed error was −1.34 g (95% CI: −4.68 to 2.00; p = 0.447), with no evidence that signed error varied systematically with served portion weight across the evaluated foods (B=−0.011 g/g; p = 0.703). The 95% Bland–Altman limits of agreement ranged from −33.44 to 30.76 g.

**Conclusions:**

The Smart24Food atlas is an open-access resource integrating photographic portion references with nutrient-composition information for foods and recipes consumed in Argentina. Under controlled direct-observation conditions, participants showed high retrieval and exact-match performance, with no evidence of systematic mean bias in visual portion matching. Further studies in more diverse populations and routine dietary-assessment contexts are needed.

## Introduction

Accurate assessment of dietary intake is essential for characterizing nutritional status and investigating diet–disease relationships across the life course, including the development and prevention of non-communicable diseases (NCDs) [1–3]. Unhealthy dietary habits are among the lifestyle factors that impact morbidity and mortality. According to the World Health Organization (WHO), nearly 80% of deaths in Argentina are attributed to NCDs, and poor dietary habits are a key modifiable lifestyle-associated risk factor for many chronic diseases globally [4–6]. Therefore, the availability of reliable tools for assessing food consumption is fundamental to identifying dietary behaviors in nutritional research, clinical practice, and public health planning.

One of the challenges in dietary assessment is accurately estimating portion sizes, a critical source of error that can lead to under- or overestimation of nutrient and energy intake [7]. To minimize these limitations, visual aids have been developed, including 3D food models, household utensils, and food photographs, which help participants recall and correctly identify the foods consumed [8].

Photographic food atlases have proven effective in minimizing errors in portion size estimation and facilitating data collection [9]. These nutritional assessment aids have been applied in various epidemiological and clinical research settings. For instance, a photographic food atlas was used in a dietary evaluation to predict the risk of gestational diabetes [10]. Another study employed a food atlas in 24h recalls to investigate the relationship between dietary zinc intake, plasma fatty acid profile, and desaturase activity in dyslipidemic subjects [11].

In recent years, several food atlases have been developed, adapted to different cultural contexts, such as in the United Arab Emirates [8], the Eastern Mediterranean [12], China [13], Malawi [14], Venezuela [15], Sri Lanka [16], Greece [17], the Balkan Region [7], Japan [18], and Ecuador [19], among others. These instruments are practical and effective for improving portion-size estimation while reflecting the distinctive dietary patterns of these populations. Since some dietary habits, recipes, and foods are region-specific, it is necessary to design dietary assessment tools appropriate for the local context [9,20].

A photographic food atlas that integrates locally relevant foods and recipes with nutrient composition data may help reduce practical barriers to using visual portion-size references in research and health education. An open-access web-based interface also allows the resource to be accessed from internet-enabled devices without requiring proprietary software. Thus, this study aimed to develop an open-access digital atlas of foods and recipes, integrating portion-size photographs and nutrient composition data, and to describe its performance for food and recipe retrieval and visual portion-size matching under controlled direct-observation conditions in a university setting in Argentina.

## Methods

### Structure and development of the tool

**Fig 1:**
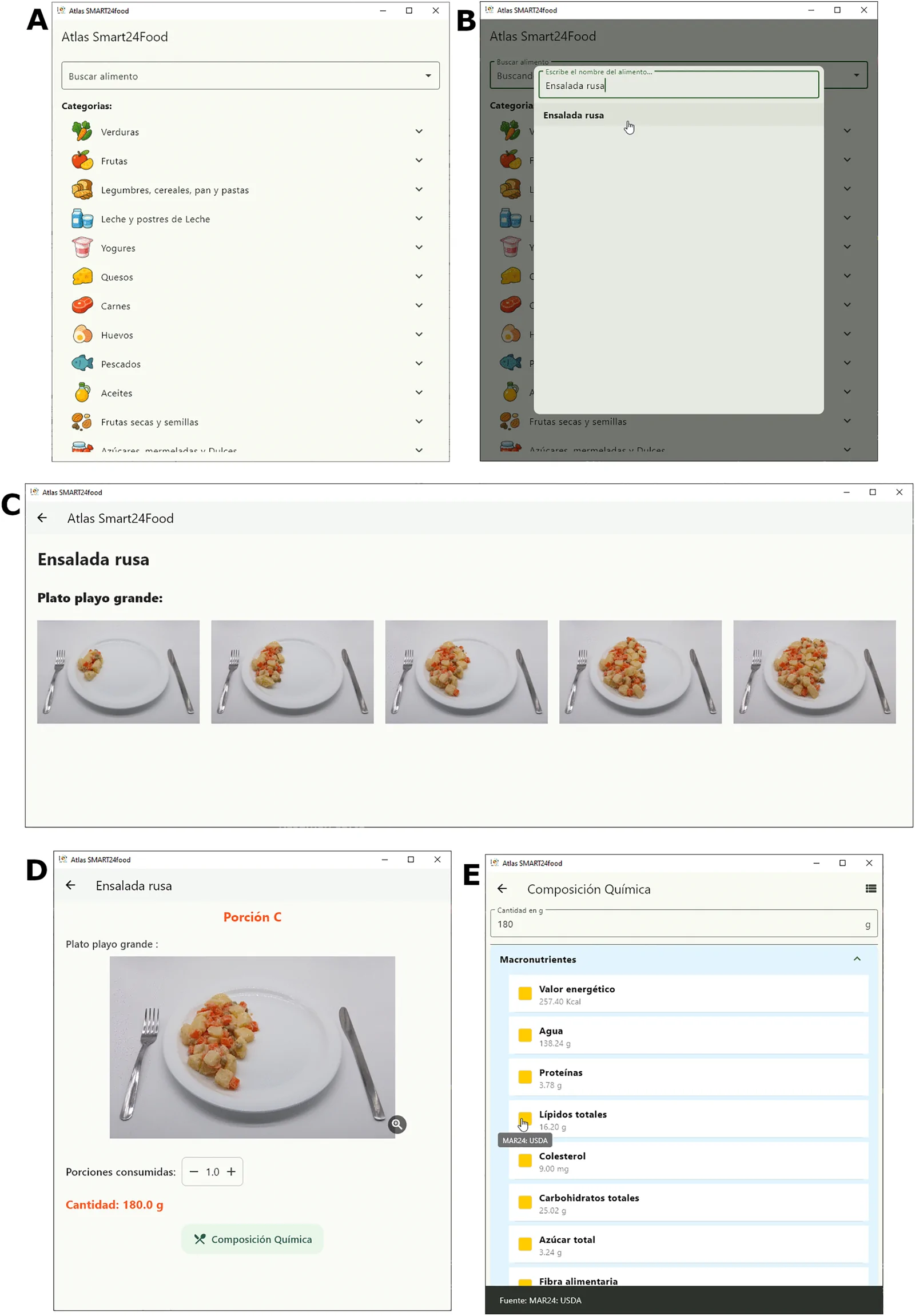
Interface and functional sections of the food atlas. **(A)** Main screen displaying food categories; **(B)** Atlas search module; **(C)** Sliding menu for portion size selection; **(D)** Presentation of the selected portion, including image zoom option, portion counter, and quantity of foods in grams; **(E)** Food nutrient display module in categorized view.

The digital interface of the photographic food atlas was developed using Flutter, a framework that enables consistent interface design and performance across various digital platforms [21]. Given that the atlas manages a significant volume of items (S1 Table), foods were organized into 26 categories defined according to nutritional and dietary similarities [22], allowing structured navigation through 998 distinct foods: vegetables; fruits; legumes, cereals, bread, and pasta; milk and dairy desserts; yogurts; cheeses; meats; eggs; fish; oils; nuts and seeds; sugars, jams, and sweets; candies and chocolates; fats; savory snacks; dressings and condiments; industrial broths and soups; industrial desserts and ice cream; salts; sugar-sweetened beverages; sugar-free beverages; alcoholic and energy drinks; natural or minimally processed fruit drinks without added sugar; infusions; fast food items; and nutritional supplements (Fig 1A). These foods were represented through 1,052 presentation-specific items. The larger item count reflects multiple visual/presentation-specific entries for some foods (e.g., sliced raw carrots versus julienne presentation).

An intuitive search box was included in the food atlas. This search option filters the food list alphabetically in Spanish as the user enters characters in real-time, accounting for food synonyms where applicable. To support flexible food retrieval, the search function was designed to be unaffected by word order, accents, or capitalization, accepting partial matches with a threshold of up to 70% of the typed word to optimize accessibility and reduce food retrieval errors (Fig 1B).

**Fig 2:**
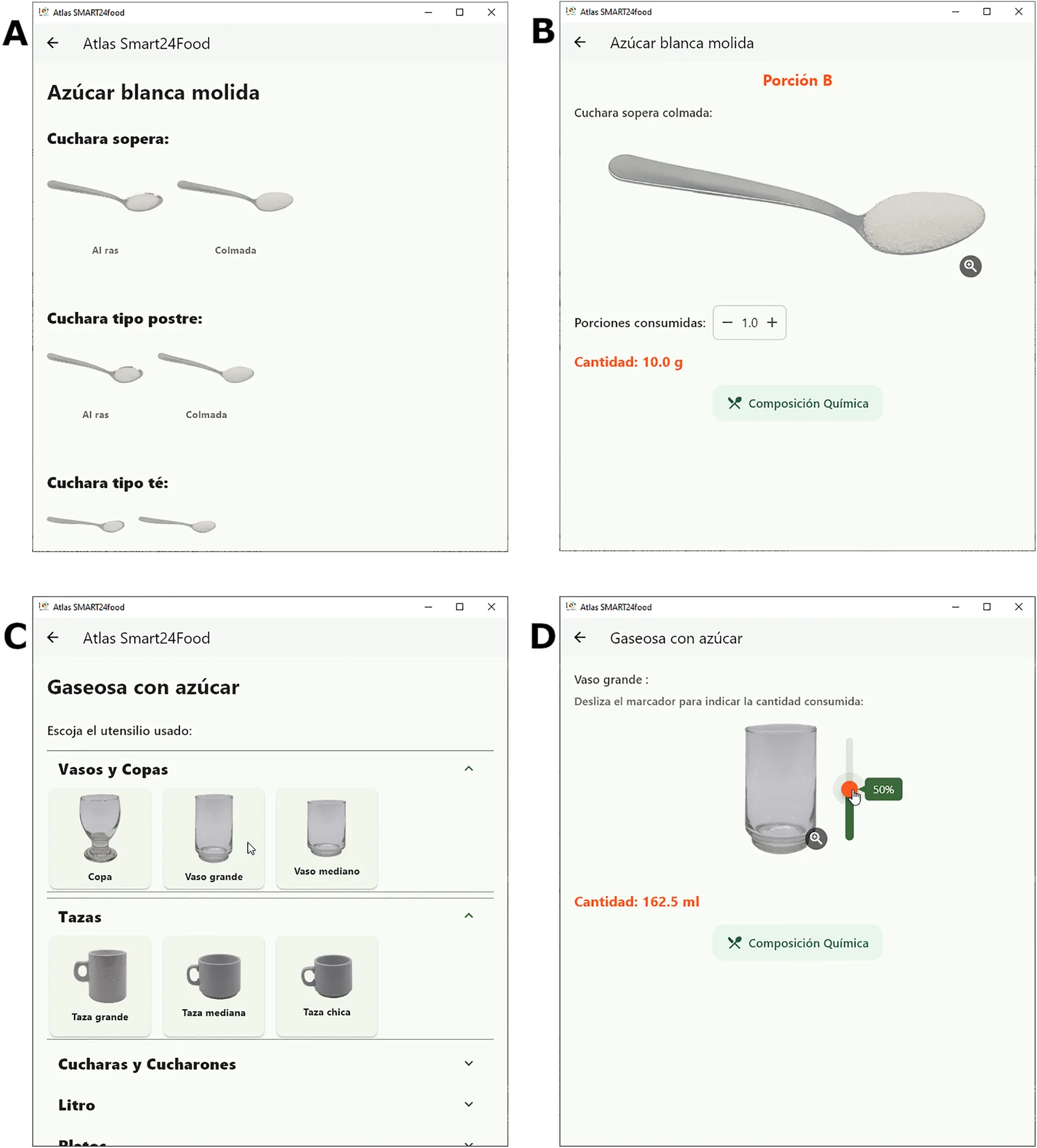
The set of measuring utensils in the atlas. **(A)** Different spoon sizes with level and heaping portions; **(B)** Selected portion, with image zoom option, portion counter, and quantity in grams; **(C)** Selection of beverage utensils (glasses, cups and mugs); **(D)** Sliding marker to indicate the amount of beverage consumed in milliliters.

The atlas also includes utensils commonly used in Argentina, such as flat plates, deep plates, cups, glasses, spoons, and ladles, as reported elsewhere [23]. These utensils aid visual identification of foods and portion-size estimation (Figs 1C, 2A, 2C, S1-S4).

The tool includes quick export buttons that let you copy nutritional information for transfer to other programs (e.g., spreadsheets), providing easy access to the nutrient composition of foods and recipes. Data are copied according to the format of the selected application view, either a categorized view of macronutrients, vitamins, minerals, and other nutrients or a flat view containing the complete list of nutrients (Fig 1E).

Portion liquid quantification was denoted using photographs of common liquid utensils (e.g., glasses and cups), indicating the corresponding milliliters (Fig 2C), combined with an interactive slider that allows users to indicate the amount consumed (Fig 2D) [23,24].

### Selection of foods, recipes, and portions

The selection of foods, recipes, portions, and utensils was mostly based on data collected by our research team in a previous study [23]. Briefly, a total of 1,165 24-hour dietary recalls were collected in 16 provinces throughout Argentina, and the data were processed, resulting in the identification and selection of common foods, recipes, portions, and utensils [23]. Additionally, other foods mentioned in the Argentine food composition database, the Food Analysis and Registry System 2 (SARA2), were included [22]. The photographed portion ranges and household measures were selected from the portions observed in those 24-h dietary recalls and were intended to span commonly reported amounts. The number of photographed portion options varied by food or recipe depending on their relevant presentations and portion range.

### Preparation of food servings and photography

Foods were purchased at various Argentine markets and prepared according to the required presentation (e.g., cubed, sliced, or whole) and, when necessary, cooked (boiled, sautéed, baked, or fried). Standardized recipes were developed for 87 dishes of the MAR24 database [23]. All raw ingredients were weighed according to the amounts established for each preparation. Recipes were then prepared, registering the type and duration of cooking. Once completed, the final product was weighed to calculate the nutritional composition of served portions. Finally, the food was portioned, then individually weighed and photographed with the corresponding utensils.

To ensure precision and reproducibility in image capture, a standardized protocol was implemented throughout the study. Initially, the first portion of the food was placed in the corresponding utensil, and additional content was gradually added, with each increment weighed and photographed to obtain measurements from smallest to largest. For foods measured by unit, such as bananas, an image of each size (small, medium, and large) was captured and associated with its respective weight. Packaged food items were also included in the atlas (n = 135).

All weighing and photography took place at the Adventist University of River Plate laboratory of nutrition and dietetics, in the Health Sciences building, in Libertador San Martín, Entre Ríos, Argentina, using high-precision digital scales (10 g to 10,000 g range; and 0.5 g to 10 g range). Images were taken at a 45° angle for general utensils and at a 90° angle for liquid utensils, at a distance of 55 cm from the center of the utensil [7,13,16]. Reference objects, such as a fork and a knife, were included in each image, and both the background and the tableware were white [7,20] (S5 Fig).

For stability and precision, a tripod (model NAXT100, Naxido, Argentina) was used, with an adjustable height from 52 cm to 150 cm and full mobility (vertical, horizontal, angular, and pivoting), featuring a built-in bubble level. Lighting was provided by a 50 cm x 50 cm x 50 cm professional portable light box (Gadnic, Argentina), equipped with two panels of 50 continuous LED lights (20 watts). The camera used was a 50 MP Samsung Galaxy M23, configured in PRO mode with ISO 100, a shutter speed of 1/750, flash disabled, and a 3:4 aspect ratio [20].

### Food image processing

Backgrounds of images depicting the set of measuring utensils (such as spoons, ladles, glasses, cups, and mugs) were removed using digital editing techniques to facilitate integration into the atlas and achieve a clean visualization (Fig 2A, 2B). These images were saved in PNG format to preserve transparency without significant quality loss.

The food images captured in the nutrition laboratory comprised 3,138 photographs, each with a resolution of 96 dpi and dimensions of 3,060 x 4,080 pixels, with an average file size of 3 MB [13]. These were subsequently centered, cropped to a 2,880 x 1,840-pixel frame, and stored in  .jpg format.

In the final stage, image compression was applied to optimize storage without limiting visual quality [25]. This procedure resulted in an average file size of approximately 0.67 MB, with the complete image set (n = 3,273) totaling 359 MB. This ensured homogeneous, high-quality visualization across the complete image set.

To optimize the use of the images, when foods shared similar visual characteristics, the same photograph and recorded weight were reused for their modified versions, preserving the visual representation while assigning different nutritional information. For instance, “Canned peaches” photography was used to represent “Canned peaches in light syrup”, assigning the corresponding nutritional composition of the light version (S1 Table). This was applied to 153 food images.

### Nutrient composition

The food nutritional profile was determined using an ordered approach based on three primary sources. Firstly, the nutrient composition of foods was selected from the Argentine chemical composition table of SARA2 [22], which included information from Argenfoods [26], United States Department of Agriculture (USDA) [27], British Table [28], and the USDA Food and Nutrient Database for Dietary Studies (FNDDS) [29]. Then, nutrients and foods not included in the SARA2 tool were selected from the MAR24 food chemical composition table [23].

Because both SARA2 [22] and MAR24 [23] incorporate USDA food-composition data [27,29], the USDA databases were consulted directly when information was unavailable in the Argentine sources. In the Smart24Food tool, each nutritional value can be identified through a small, color-coded indicator displayed alongside each compound in the nutrient list (Fig 1E and S2 Table).

The nutrient composition of recipes was estimated using the Food and Agriculture Organization of the United Nations (FAO) recipe analysis methodology [30]. First, nutritional data for each raw ingredient were collected based on weights and food composition tables [22,23,27]. The energy and nutrient contributions of each ingredient were then calculated based on the amount used. Once partial values were obtained, nutrient-specific retention factors were applied according to the cooking method and the final weight of the food, following the USDA retention table [31]. Finally, the recipe’s chemical composition was expressed per 100 g of prepared food, and the contribution of each portion, as defined by the photographs, was estimated.

### Direct-observation performance study

The direct-observation performance study was conducted in the university dining hall. A convenience sample of 108 young adults was recruited in this setting. Twenty representative foods and recipes [23] were selected and distributed into three groups: packaged products (n = 7), minimally processed foods (n = 7), and standardized recipes (n = 6). The items served were displayed individually, under controlled direct-observation conditions (S6 Fig). Inclusion criteria were age ≥ 18 years, Argentine nationality or residence, ability to operate an electronic device, and voluntary participation. After providing written informed consent, participants accessed the atlas on their personal device, located each presented item, and selected the atlas image that best matched the displayed product or portion. A researcher observed the task only to record the selected option and whether the intended food or recipe could be retrieved [12,19,32].

Two task types were prespecified. For the seven packaged-product tasks, an exact match required selection of the correct product and presentation, including the corresponding package size when more than one presentation was available. For the 13 visual portion-matching tasks, each served portion was deliberately prepared to correspond to one of the discrete photographed portions and its associated weight in the atlas. An exact match would require selection of that specific photographic food and portion size. Food-retrieval success was recorded separately whenever the intended item was effectively identified in the atlas. The evaluated foods were presented using serving utensils represented in the atlas so that visual matching occurred under conditions consistent with the atlas images.

The tool-specific usability outcomes, task-completion time, satisfaction, and perceived difficulty were not separately measured. Therefore, the study is described in terms of food retrieval and exact-match performance rather than general usability.

### Statistical analysis

Food-retrieval success, overall exact-match performance, food-specific exact-match performance, participant-level totals, and participant demographics were summarized descriptively. The binary exact-match outcome was analyzed inferentially using cross-classified logistic mixed-effects models.

#### Exact-match analyses and sample-size determination.

An intercept-only cross-classified logistic mixed-effects model was initially fitted to the binary exact-match outcome to quantify variability attributable to participants and foods. The model included random intercepts for participant and food, accounting for the 20 repeated tasks completed by each participant and the 108 evaluations performed for each food.

Food category (packaged product, minimally processed food, standardized recipe) was then evaluated as a fixed effect. The adjusted model additionally included sex, age, and nutrition-related study or work, while retaining random intercepts for participant and food. Pairwise food-category contrasts were adjusted using the Holm method. Educational level and field of study or work were summarized descriptively but could not be modeled inferentially because of sparse categories, including only one participant with postgraduate education. A complementary model replaced food category with task type (packaged-product selection versus visual portion-size matching). Category and task type were not entered simultaneously because task type was structurally determined by food category.

The sample size was calculated to provide adequate precision for the descriptive assessment of exact-match performance across the prespecified direct-observation tasks. Assuming a conservative participant-level standard deviation of 15 percentage points, a two-sided 95% confidence interval, and a target confidence-interval half-width of 3 percentage points, the minimum required sample was 97 evaluable participants, n=(1.96 × 0.15/0.03)^2^ = 96.04. The target sample was rounded to 100 participants, and recruitment was extended to 108 participants to allow for potentially incomplete or non-evaluable observations. All 108 participants completed the 20 prespecified tasks and were included in the analyses [12,13,32,33].

#### Weight-based analyses and agreement.

For weight-based analyses, signed error was calculated as the photograph-associated selected weight minus the served weight. Negative values indicated underestimation, and positive values indicated overestimation. An initial cross-classified linear mixed-effects model included random intercepts for participant and food. Because the participant-level variance was estimated at approximately zero, resulting in a singular fit, the final parsimonious model retained only a random intercept for food. This model was used to estimate the mean signed error and its 95% confidence interval.

To explore whether signed error varied according to portion size, a second linear mixed-effects model included served weight as a fixed effect and food as a random intercept. Because the served weight was constant within each food, this analysis assessed the association between signed error and served portion weight across the 13 evaluated foods.

For the 13 visual portion-matching tasks (1,404 participant–food observations), a repeated-measures Bland–Altman analysis was used descriptively to display the difference between the photograph-associated selected weight and the served weight against their pairwise mean. Repeated observations within participants were treated as nested, and the figure reports the mean difference and 95% limits of agreement. Inferential conclusions regarding mean bias and change in bias with served weight were based on the mixed-effects models described above. Mixed-effects analyses were performed in JAMOVI 2.7 using GAMLj3. The Bland–Altman analysis and figure were generated in R 4.6.1 using SimplyAgree 0.3.0. All inferential tests were two-sided with α = 0.05 and 95% confidence intervals.

### Ethical considerations

Participants were included in the study after accepting the invitation to participate and providing informed consent. All procedures were conducted in accordance with the international ethical standards in the Declaration of Helsinki for research involving human subjects. This study was reviewed and approved by the Research and Ethics Committee of the Adventist University of River Plate School of Medicine (Resolution No. 95/23), which is affiliated with the National Registry of Health Research (Registry No. 000237) of the Argentine Ministry of Health. The recruitment period for this study began on September 30, 2024, and ended on February 12, 2026. The authors had no access to information that could identify individual participants.

## Results

The food atlas included 3,273 photographs representing 998 distinct foods through 1,052 presentation-specific items. Each food features a nutritional profile with up to 53 available elements. The food atlas may be accessed at the following link: https://smart24food.uap.edu.ar/atlas/. The direct-observation performance evaluation included 108 participants, mostly young adults. Their sociodemographic characteristics are shown in Table 1.

**Table 1:** Sociodemographic characteristics of study participants (n = 108).

Variables	Values	
	n	(%)
Sex		
Male	47	43.5
Female	61	56.5
Age range (mean = 22.7, SD = 6.08)		
18-19	31	28.7
20-21	28	25.9
22-24	30	27.8
25-51	19	17.6
Educational level		
High school graduate	22	20.4
Technical/tertiary degree	75	69.4
University degree	10	9.3
Postgraduate degree	1	0.9
Field of study or work		
Economic sciences	14	13
Humanities	28	25.9
Health sciences	53	49.1
Theology	8	7.4
Other	5	4.6
Nutrition-related study or work		
No	90	83.3
Yes	18	16.7

Table 2 presents the nutritional composition of the “Russian salad” recipe, which includes boiled potatoes and carrots, fresh peas, mayonnaise, and salt.

**Table 2:** Example of nutrient report for 100 grams of the “Russian salad” standardized recipe.

Category/Nutrient	Value	Unit
Energy value	143	Kcal
Macronutrients		
Water	71.2	g
Protein	2.1	g
Total lipids (fat)	9	g
Cholesterol	5	mg
Total carbohydrates	13.9	g
Total sugars	1.8	g
Dietary fiber	2.4	g
Alcohol	0	g
Animal protein	0.1	g
Vegetable protein	2.0	g
Fatty acids		
Saturated	1.41	g
Monounsaturated	1.99	g
Polyunsaturated	5.35	g
18:2 cis-linoleic	0.07	g
18:3 cis-alpha-linolenic (ALA)	0.01	g
20:5 n-3 eicosapentaenoic (EPA)	0	g
22:6 n-3 docosahexaenoic (DHA)	0	g
Omega-3 (22:5 DPA)	0	g
Vitamins, minerals, and others		
Minerals		
Ash	1.51	g
Sodium	237	mg
Potassium	334	mg
Calcium	17	mg
Copper	0.09	mg
Phosphorus	52	mg
Iron	0.72	mg
Magnesium	19	mg
Zinc	0.32	mg
Manganese	0.15	mg
Water-soluble vitamins		
Niacin	0.94	mg
Folate, DFE	17	µg
Thiamin	0.08	mg
Riboflavin	0.04	mg
Vitamin B-12	0	µg
Vitamin C	14	mg
Pantothenic acid (B5)	0.28	mg
Vitamin B-6	0.22	mg
Fat-soluble vitamins		
Vitamin A, RAE	154	µg
Retinol	2	µg
Vitamin D (D2/D3)	1	µg
Vitamin E (alpha-tocopherol)	0.55	mg
Vitamin K (phylloquinone)	27.07	µg
Other bioactive compounds		
Choline	16.6	mg
Caffeine	0	mg
Theobromine	0	mg
Carotenoids		
beta-Carotene	1,524	µg
alpha-Carotene	615	µg
beta-Cryptoxanthin	0	µg
Lycopene	0	µg
Lutein/zeaxanthin	210.97	µg

### Direct-observation performance

The intercept-only cross-classified logistic mixed-effects model converged successfully. Significant variability was observed both between participants and between foods. The participant-level random-intercept variance was 0.363 (SD = 0.602; 95% CI for the SD: 0.376–0.837; likelihood-ratio χ^2^ = 14.1, p < 0.001), whereas the food-level variance was 2.318 (SD = 1.522; 95% CI for the SD: 1.097–2.248; likelihood-ratio χ^2^ = 401.9, p < 0.001). The substantially greater food-level variance indicated that exact-match performance differed more strongly across foods than across participants. The model had a conditional R^2^ of 0.449.

Exact-match proportions were descriptively lower for minimally processed foods than for packaged products and standardized recipes. However, in the cross-classified logistic mixed-effects model with random intercepts for participant and food, food category was not significantly associated with the probability of an exact match (Wald χ^2^(2) = 4.71, p = 0.095). Compared with packaged products, minimally processed foods showed lower estimated odds of an exact match, although the confidence interval included the null value (OR = 0.23, 95% CI: 0.05–1.02; unadjusted p = 0.053; Holm-adjusted p = 0.159). No evidence of a difference was observed between standardized recipes and packaged products (OR = 0.92, 95% CI: 0.19–4.37; p = 0.913). None of the pairwise comparisons remained statistically significant after Holm correction.

In the cross-classified logistic mixed-effects model including food category and participant characteristics, sex was associated with exact matching: female participants had higher odds of an exact match than male participants (OR = 1.55, 95% CI: 1.08–2.24, p = 0.019). No associations were observed for age (OR=0.98 per year, 95% CI: 0.94–1.02, p = 0.329) or nutrition-related study or work (OR=1.22, 95% CI: 0.63–2.37, p = 0.562). Food category was not significantly associated with exact matching (χ^2^(2)=4.70, p = 0.095), and none of the pairwise comparisons remained statistically significant after Holm adjustment. Significant residual variability remained between participants (variance = 0.299, p = 0.001) and, particularly, between foods (variance = 1.812, p < 0.001). In a complementary model replacing food category with task type, no significant difference in exact-match probability was observed between visual portion-matching tasks and packaged-product selection tasks (OR = 0.42, 95% CI: 0.10–1.77, p = 0.238). Estimates for sex, age, and nutrition-related study or work remained essentially unchanged. Inferential comparisons were based on the cross-classified mixed-effects models reported above. Table 3 summarizes the fixed-effect estimates from the adjusted model.

**Table 3:** Fixed effects from the adjusted cross-classified logistic mixed-effects model for exact matching.

Predictor	Contrast	OR	95% CI	p-value	Holm-adjusted p- value
Food category	Minimally processed vs packaged	0.23	0.05–1.02	0.053	0.159
Food category	Standardized recipe vs packaged	0.92	0.19–4.37	0.913	0.913
Sex	Female vs male	1.55	1.08–2.24	0.019*	
Age	Per 1-year increase	0.98	0.94–1.02	0.329	
Nutrition-related study or work	Yes vs no	1.22	0.63–2.37	0.562	

Food-category omnibus Wald χ^2^(2)=4.70, p = 0.095. Random-intercept variances in the adjusted model were 0.299 for participant and 1.812 for food. Holm adjustment was applied only to pairwise food-category contrasts.

The initial cross-classified linear mixed-effects model yielded an approximately zero participant-level variance and a singular fit; thus, the final model retained food as the only random-intercept factor. The estimated mean signed error was −1.34 g (95% CI: −4.68 to 2.00; p = 0.447), indicating no evidence of a systematic mean bias toward under- or overestimation. Variability between foods remained appreciable (variance = 35.5 g^2^; SD = 5.96 g).

In the second linear mixed-effects model, there was no evidence that signed error varied systematically with served portion weight across the evaluated foods (B=−0.011 g per gram, 95% CI: −0.064 to 0.043; F(1,11)=0.153, p = 0.703). Thus, larger served portions were not associated with a systematic tendency toward greater under- or overestimation under the evaluated conditions.

#### Food retrieval and overall exact matching.

Across the 2,160 participant–food tasks, 1,869 selections were exact matches, corresponding to a descriptive overall exact-match proportion of 86.5%. Food retrieval was successful in 2,156 of 2,160 tasks (99.81%), and only 4 attempts to correctly identify the items were unsuccessful. Retrieval success was analyzed separately from exact matching and should not be interpreted as a general usability score.

Educational level and field of study or work are reported descriptively in Table 1. They were not used for inferential subgroup conclusions because several categories were sparse (university degree, n = 10; postgraduate degree, n = 1; and some fields of study/work had small counts).

#### Food-specific exact matching.

Exact-match counts and percentages were summarized descriptively for each of the 20 evaluated items (Table 4). The seven packaged-product tasks were items 1, 3, 5, 7, 9, 13, and 15; the remaining 13 tasks involved visual portion matching and therefore also contributed to the continuous weight-error analyses.

**Table 4:** Descriptive exact-match performance and weight-error measures by evaluated food or recipe.

		Exact match				Weight-error measures		
Food		Yes	(%)	No	(%)	Mean Error (g)	MAE (g)	MAPE (%)
1	Chocolate alfajor, mousse-type	106	98.1	2	1.9			
2	Raw chopped lettuce	102	94.4	6	5.6	0.39	0.39	5.56
3	Chocolate bar, Kinder-type	104	96.3	4	3.7			
4	Tomato, lettuce, and onion salad	93	86.1	15	13.9	2.50	6.67	4.76
5	Milk chocolate	106	98.1	2	1.9			
6	Vegetable soup	105	97.2	3	2.8	3.06	3.06	2.78
								
7	Triple alfajor filled with dulce de leche	108	100	0	0			
8	Raw sliced cucumber	35	32.4	73	67.6	-16.33	17.56	17.91
9	Chocolate with almonds	79	73.1	29	26.9			
10	Sunflower seeds	99	91.7	9	8.3	0.20	0.27	6.20
11	Raw oats	97	89.8	11	10.2	0.52	2.44	4.44
12	Vegetable and meat soup	105	97.2	3	2.8	-1.02	3.06	1.39
13	Peanut paste-based dessert	100	92.6	8	7.4			
14	Boiled white rice	78	72.2	30	27.8	-0.97	10.05	9.13
15	Candy-coated chocolate	90	83.3	18	16.7			
16	Raw grated carrot	107	99.1	1	0.9	0.19	0.19	0.62
17	Carrot, lettuce, and tomato salad	96	88.9	12	11.1	5.42	5.42	5.70
18	Raw sliced tomato	51	47.2	57	52.8	-11.34	13.66	13.01
19	Vegetable and cereal soup	104	96.3	4	3.7	4.07	4.07	3.70
20	Meat stew	104	96.3	4	3.7	-4.07	4.07	1.85
	Total	1869	86.5	291	13.5			

Continuous weight-error measures were calculated only for the 13 visual portion matching tasks. They were not calculated for the seven packaged-product selection tasks (items 1, 3, 5, 7, 9, 13, and 15), for which the outcome was selection of the correct commercial product/presentation. Mean signed error = selected photograph-associated weight − served weight; MAE = mean absolute error; MAPE = mean absolute percentage error.

Food-specific exact-match performance varied substantially. The lowest raw exact-match proportions were observed for raw sliced cucumber (item 8; 32.4%), raw sliced tomato (item 18; 47.2%), boiled white rice (item 14; 72.2%), and chocolate with almonds (item 9; 73.1%). Most of the other items had exact-match proportions above 95%. Among the 13 visual portion-matching tasks, the largest negative mean signed errors were observed for raw sliced cucumber (−16.33 g) and raw sliced tomato (−11.34 g). These food-level values are descriptive and complement the mixed-effects analyses, which account for repeated observations and food-level heterogeneity.

#### Participant-level exact matching.

Participants achieved a mean of 17.3 out of 20 tasks (SD = 1.67), with a median of 18 and a range of 11–20 exact matches. These participant-level summaries are descriptive and do not imply equivalent performance across demographic or educational subgroups.

#### Weight-based error and Bland–Altman description.

**Fig 3:**
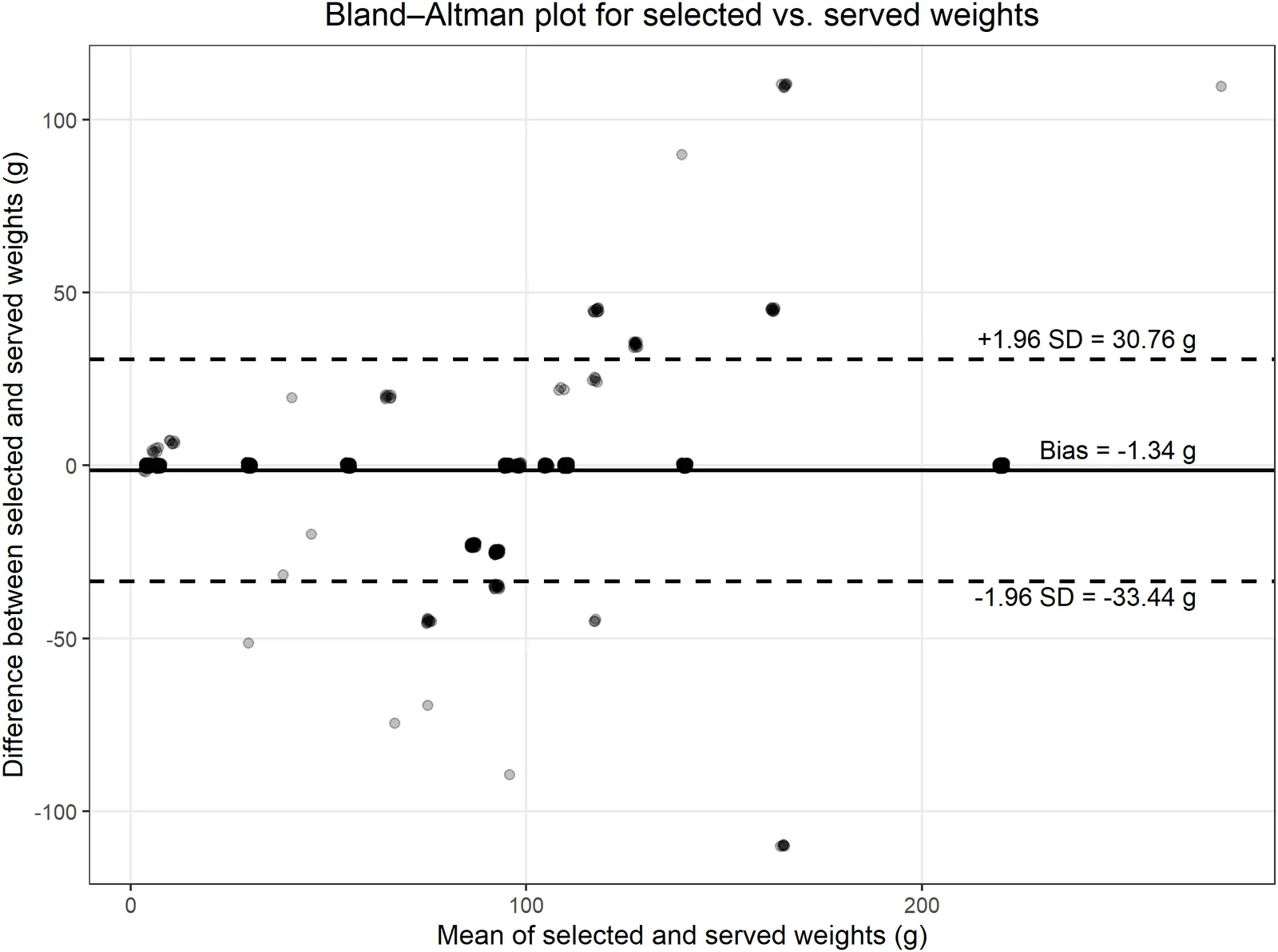
Bland–Altman plot of photograph-associated selected weight and served weight. The plot includes 1,404 participant–food observations from 108 participants across 13 visual portion-matching foods/recipes. The x-axis shows the mean of the selected and served weights (g), and the y-axis shows the signed difference (selected minus served weight, g). The solid line indicates the mean difference (−1.34 g), and the dashed lines indicate the descriptive 95% limits of agreement (−33.44 to 30.76 g). Points were slightly jittered for visualization because many observations overlapped.

Across the 1,404 visual portion-matching observations, the repeated-measures Bland–Altman analysis yielded a mean difference of −1.34 g and descriptive 95% limits of agreement from −33.44 to 30.76 g (Fig 3).

The mixed-effects model reported above provided the inferential estimate for mean signed error (−1.34 g, 95% CI: −4.68 to 2.00; p = 0.447), and the served-weight model showed no evidence that signed error varied systematically with served weight across the evaluated foods (B = −0.011 g per gram, 95% CI: −0.064 to 0.043; p = 0.703).

## Discussion

The Smart24Food atlas expands the digital infrastructure available for dietary assessment in Argentina by combining a comprehensive photographic collection with food retrieval, portion-specific images, household utensils, standardized recipes, and nutrient composition information into a single open-access resource. In the controlled direct-observation study, participants retrieved the intended food or recipe in 99.81% of tasks and achieved an exact match in 86.5% of the 2,160 participant–food observations. The mixed-effects analyses add an important qualification to these descriptive results: performance varied more substantially across foods than across participants, whereas broad food category and task type were not significantly associated with exact matching after the crossed participant and food structure was modeled. Among the 13 visual portion-matching tasks, the mean signed error was −1.34 g, with no evidence that the average error differed from zero or changed systematically with served portion weight. These findings suggest that difficulty in exact matching under direct-observation conditions was more strongly related to heterogeneity across specific foods and presentations than to participant-level variability.

In Argentina, the Smart24Food atlas is complementary to rather than redundant with existing resources. The Digital Photographic Atlas of Argentine Foods (AFDAA) contains 292 photographs organized into 103 food and dish series and was developed to support portion quantification in the Second National Survey of Nutrition and Health in Argentina [32]. The Smart24Food atlas has a broader visual scope, with 3,273 photographs representing 998 distinct foods through 1,052 presentation-specific items, and it links these representations to nutrient-composition information. Its food, recipe, portion, and utensil content was largely informed by the MAR24 study, which accumulated 1,165 24-hour recalls from adults in different Argentine regions and used these observations to expand a locally relevant food and recipe database [23]. This approach is consistent with the methodological principle that food atlases should be grounded in foods and portion ranges consumed by the target population [20]. Similar integration of photographic aids with larger dietary-assessment infrastructures has been pursued in the Balkan region, where the atlas was incorporated into a food-composition database and the Diet Assess & Plan platform [7]. In China, an atlas containing 799 photographs of 303 foods was developed to improve retrospective food-quantity estimation [13]. The Smart24Food atlas extends this model by placing photographic portions, multiple visual presentations, standardized recipes, utensils, search functions, and nutrient data into an openly accessible digital environment.

The 86.5% exact-match proportion is particularly informative when interpreted against studies that used different scoring rules. In the Balkan validation study, exact selection of the correct photograph averaged 60.2% among photo series considered acceptable, whereas more than 98% of selections were correct or adjacent [7]. In Greece, approximately 90% of assessments were classified as correct or adjacent, and the investigators noted that earlier perception studies often reported only about 50% exact selections and about 70% correct-or-adjacent selections [17]. In our study, the criterion was deliberately strict. Visual portion tasks success required the selection of a specific photograph whose associated weight equaled the served portion. For packaged products, it required the correct identification of the intended commercial product/presentation and size. Thus, the observed 86.5% represents a demanding fixed-image criterion rather than a tolerance band around the reference value.

The finding is also compatible with the Argentine AFDAA study, where 57% of evaluated photo sets had a mean percentage difference below 20%, and 63% had at least half of observations within ±30% of the real weight [32]. Considered together, this evidence indicates that photographic aids can perform well at the group level, but the apparent magnitude of “accuracy” depends strongly on whether studies score exact photographs, adjacent photographs, percentage-weight tolerances, or continuous gram differences.

The crossed mixed-effects results in our study indicated that food identity was a larger source of heterogeneity than participant identity, consistent with previous studies reporting marked food-specific variation in photographic portion-size estimation. Raw sliced cucumber and raw sliced tomato produced the largest negative mean signed errors among the visual portion tasks, while boiled rice had a relatively low exact-match proportion despite a small mean signed error. Comparable food-specific difficulties have been described elsewhere. In Malawi, digital photographs performed relatively well for a single-unit food such as banana, whereas amorphous foods and stews were more difficult to estimate and the staple “nsima” food was systematically underestimated [14]. Among Japanese adults, errors varied markedly across the 14 tested foods, from a 29.8% underestimation for curry sauce to a 34% overestimation for margarine [33]. The UAE study likewise found both substantial underestimation and overestimation, depending on the food item, with wide individual variability [8]. These patterns support the interpretation of our results, where some of the weaker items (e.g., raw sliced cucumber) are more difficult to estimate, but this is not evidence that an entire food category is intrinsically unsuitable for photographic estimation.

The weight-based results found in our study support the range reported for a photographic portion-size aid and illustrate why group-level bias and individual-level agreement should be considered separately. The mean signed error of −1.34 g was close to zero, and the mixed-effects confidence interval (−4.68 to 2.00 g) provided no evidence of a systematic average tendency toward under- or overestimation. The descriptive Bland–Altman limits of agreement (−33.44 to 30.76 g), however, show that individual selections can deviate considerably even when the average bias is small. A similar distinction appears in earlier studies. Tueni et al. reported negligible mean bias when participants directly viewed presented Lebanese foods (approximately −0.2 g) and a larger negative bias when portions were recalled the following day (approximately −6.3 g) [34]. In southern Nepal, the overall mean error was −4.5%, but specific foods differed substantially. Rice and “dal” food were underestimated by 14.1% and 34.5%, respectively, whereas vegetable curry was overestimated by 20.8%. The authors also emphasized wide limits of agreement despite relatively small average energy error [35]. The Young Person’s Food Atlas in the United Kingdom found mean portion estimates within 7% of weighed records but similarly reported wide limits of agreement at the individual level [36]. These comparisons show that a near-zero mean difference should not be interpreted as uniformly precise performance for every participant or every food.

The Smart24Food atlas differed from previous studies in the pattern of error across portion-size estimation. Across the 13 evaluated foods, the model using served weight as a predictor provided no evidence that signed error varied systematically with served portion weight. Larger served portions were therefore not associated with a systematic shift toward greater under- or overestimation under the evaluated conditions. This differs from the frequently described “flat-slope” phenomenon, in which small portions tend to be overestimated and large portions underestimated. The Greek digital atlas observed this pattern [17], and Szenczi-Cseh et al. similarly reported overestimation of small portions and underestimation of large portions in a perception experiment, with still larger errors when memory was introduced [37]. Japanese data also showed increasing variability at larger serving sizes for many foods [33]. In our study, results suggest that within the 13 tested foods and their fixed-served portions, there was no consistent directional change in signed error as weight increased. It does not imply that the variance of errors is constant across all portion sizes or that a flat-slope effect could not emerge in a larger set of foods.

Participant characteristics showed limited associations with performance. In the adjusted analysis, female participants had higher odds of an exact match than male participants, whereas age and nutrition-related study or work were not associated with performance. Overall, previous food-atlas studies have generally reported little or no association between portion-size estimation performance and sociodemographic characteristics, including sex, age, educational level, place of residence, or BMI [8,12,14,19,33]. Therefore, the association observed with sex in the present study should be interpreted cautiously, as a study-specific finding that requires replication rather than as evidence of a consistent sex-related difference in visual portion perception. Educational level and field of study were not examined inferentially because of sparse categories.

The high food-retrieval rate addresses a different component of performance from portion perception. Only four of 2,160 tasks failed to retrieve the intended food or recipe, suggesting that the search and navigation structure generally allowed participants to locate the target item under direct-observation conditions. This finding is relevant because digital dietary systems depend not only on portion images but also on the ability to efficiently find the correct, searched food.

Packaged products represent a distinct visual-identification task from foods served as portions. Photographic atlases have usually incorporated market-specific representations of commercial products. For example, the Sri Lankan food atlas used market surveys to identify different brands and container sizes and included guide photographs representing different brands of commercially available products, including ready-to-serve milk products [16]. Similarly, packaged items have been treated separately in other photographic dietary-assessment systems, and their comparatively fixed presentation may facilitate recognition. In the Young Person’s Food Atlas, packaged foods such as biscuits, yoghurt, and chocolate bars were among the items showing better agreement, which the authors partly attributed to easier identification and less variation in portion size [36].

In our results, the packaged-product tasks show the importance of preserving visual scale across alternative commercial presentations. This was particularly observed for chocolate with almonds, for which participants had to distinguish the 50-g presentation from another package size of the same product, and exact-match performance was lower than for most other packaged-product tasks. The commercial images used during participant testing were not standardized in photographic scale and perspective, which may have reduced the visual information available for discriminating between package sizes. This finding suggests that, for packaged products with similar visual appearance but different commercial presentations, consistent perspective and relative-size cues may be particularly important for accurate visual identification. This is consistent with previous food-atlas approaches that have incorporated market-specific representations of commercial products and package sizes [16].

An additional feature of the Smart24Food atlas is the integration of portion-size photographs with nutrient-composition information in the same digital resource. This distinguishes the atlas from photographic tools designed exclusively to support portion-size estimation and allows the selected visual representation to be directly linked to its corresponding nutrient profile. Nutrient values were compiled preferentially from Argentine food-composition sources and supplemented, when necessary, with international databases, while standardized recipes were calculated using established food-composition and nutrient-retention procedures [27,28,30,31]. This approach increases the functional scope of the food atlas by connecting visual portion identification with quantitative dietary information. However, these nutrient profiles represent compiled or calculated values rather than direct chemical analyses of each photographed food. Consequently, periodic review and updating are necessary, particularly for commercial products whose formulations may change over time.

Several features strengthen the interpretation of these findings. Atlas content and portion ranges were grounded primarily in dietary data collected in Argentina [20,23], and laboratory photographs were obtained under standardized conditions. The direct-observation design allowed visual perception to be examined without simultaneously introducing the conceptualization and memory errors inherent to recall-based tasks [17,34,37]. In addition, the cross-classified mixed-effects analyses explicitly accounted for repeated observations within participants and repeated evaluations of foods, preventing the 2,160 task-level observations from being treated as independent measurements.

The study also has limitations that define the scope of inference. First, participants formed a convenience sample recruited in a university setting and were predominantly young adults. Thus, these results cannot be generalized to the general population. Second, the direct-observation design primarily assessed visual perception and food retrieval while the target foods remained visible. It did not reproduce the conceptualization and memory demands involved in recall-based dietary assessment. These components represent distinct sources of error in photographic portion-size estimation [17,34,37], and the performance observed under direct-observation conditions should therefore not be extrapolated to memory-based dietary assessment methods. Third, only 20 tasks were evaluated from an atlas containing many presentation-specific items, so performance of other untested foods cannot be exactly inferred from the evaluated subset, and this limitation is observed in similar studies as well. Fourth, exact matching was facilitated by the fact that the served portions corresponded specifically to one of the available atlas photographs. In some dietary assessment contexts, the consumed amount may not correspond exactly to any of the available images, requiring users to select the photograph that most closely resembles the portion consumed. Systematic reviews of portion-size aids emphasize that photograph number, spacing, food type, presentation, and the cognitive demands of the dietary method may all influence estimation error [9]. Finally, although the Bland–Altman display summarizes the dispersion of the 1,404 weight pairs, the wide range of individual errors and the food-level heterogeneity observed in the mixed models indicate that the atlas should not be characterized as uniformly precise at the individual level. These limitations also inform useful next steps for further studies.

## Conclusion

The Smart24Food atlas is an open-access digital resource that integrates portion-size photographs, household utensils, standardized recipes, and nutrient-composition information for 998 distinct foods represented through 1,052 presentation-specific items. In the evaluated sample and under controlled direct-observation conditions, food retrieval was successful in almost all tasks, and the overall exact-match proportion was adequately high. Across the 13 visual portion-matching tasks, mixed-effects analyses showed no evidence of systematic mean bias or of a systematic change in signed error with served portion weight across the evaluated foods. These findings characterize performance for the specific food-retrieval and fixed-image matching tasks studied but should not be interpreted as validation of general usability or recall-based dietary assessment. Further studies in more diverse populations and under routine dietary assessment conditions are needed to determine how this performance translates to broader applications.

## Supporting information

S1 Table List of foods included in the atlas. (PDF)

S1 Fig Utensils used for serving and food-portion estimates. (TIF)

S2 Fig Glasses and a 1-liter bottle. (TIF)

S3 Fig Mugs and cups used in the photographic atlas. (TIF)

S4 Fig Plates used in the photographic atlas. (TIF)

S5 Fig Side view of the photographic setup used, showing the angle, distance to the plate, and controlled lighting. (TIF)

S2 Table Nutrient composition data sources with their identifying colors. (PDF)

S6 Fig Food atlas evaluation process. (TIF)
