# Supplementary material for: Development of an open-access food atlas with nutrient composition and recipes in Argentina: The Smart24Food Atlas
Source: PLoS One. 2026 Sep 25;21(9):e0359331. doi: 10.1371/journal.pone.0359331 (PMC13614558; doi:10.1371/journal.pone.0359331)
Supplement: Supplementary file 1 [file pone.0359331.s001.pdf]

S1 Table. List of foods included in the atlas.

N°	Food code	Food	Reference food code	Food synonyms
1	1	Acelga, cruda		
2	1-a	Acelga picada cruda		
3	4	Acelga, hoja, hervida		
4	5	Achicoria, hoja, cruda		
5	5-a	Achicoria picada cruda		
6	6	Achicoria, hoja, hervida		
7	6-a	Achicoria picada hervida		
8	8	Morrón rojo, crudo		
9	8-a	Morrón rojo picado crudo		
10	8-b	Ají rojo crudo		
11	8-c	Ají rojo picado crudo		
12	9	Morrón rojo, rehogado		
13	10	Morrón verde, crudo		
14	10-a	Morrón verde picado crudo		
15	10-b	Morrón amarillo crudo		
16	10-c	Morrón amarillo picado crudo		
17	10-d	Ají verde crudo		
18	10-e	Ají verde picado crudo		
19	10-f	Ají amarillo crudo		
20	10-g	Ají amarillo picado crudo		
21	11	Ají verde o amarillo / morrón verde o amarillo, rehogado		
22	11-a	Morrón verde picado salteado con aceite y sal		
23	11-b	Morrón amarillo picado salteado con aceite y sal		
24	12	Ajo, crudo		
25	13	Albahaca, hoja, cruda		
26	13-a	Albahaca picada cruda		
27	16	Alcaucil, hervido		
28	17	Apio picado crudo		
29	18	Apio picado hervido		
30	19	Arveja, enlatada	21	
31	21	Arveja, fresca, hervida		
32	23	Berenjena hervida		
33	23-a	Berenjena en rodajas hervidas		
34	23-b	Berenjena en cubos hervidas		
35	25	Brócoli, crudo		
36	25-a	Brócoli picado crudo		
37	26	Brócoli, hervido		
38	26-a	Brócoli picado hervido		
39	29	Cebolla de verdeo, cruda		
40	30	Cebolla de verdeo, rehogada		
41	31	Cebolla, cruda		
42	31-a	Cebolla picada en juliana cruda		
43	31-b	Cebolla picada en cubos cruda		
44	32	Cebolla, rehogada		
45	32-a	Cebolla picada en cubos salteada con sal y aceite		
46	32-b	Cebolla picada en juliana salteada con sal y aceite		
47	33	Champignones, enlatados		
48	36	Chaucha, fresca, hervida		
49	36-a	Chaucha fresca picada hervida		
50	39	Coliflor, hervido		
51	39-a	Coliflor picado hervido		
52	42	Espárrago, hervido		
53	42-a	Espárrago picado hervido		
54	43	Espinaca, hoja, cruda		
55	43-a	Espinaca picada cruda		
56	44	Espinaca, hoja, hervida		
57	44-a	Espinaca picada, hervida		
58	47	Hakusai o col china, crudo		
59	48	Hinojo, crudo		
60	48-a	Hinojo picado en juliana crudo		
61	48-b	Hinojo picado crudo		
62	54	Jardinera, enlatada		
63	55	Jengibre		
64	55-a	Jengibre en rodajas		
65	55-b	Jengibre picado		
66	56	Kale, hoja, cruda		
67	56-a	Kale picado, crudo		
68	57	Lechuga, hoja, cruda		
69	57-a	Lechuga picada cruda		
70	59	Mandioca, hervida		Yuca, hervida
71	61	Palmitos, enlatados		
72	61-a	Palmitos picado enlatados		
73	62	Pepino, crudo		
74	62-a	Pepino en rodajas crudo		
75	63	Perejil, crudo		
76	63-a	Perejil picado crudo		
77	64	Pickles/Pepinillos, en vinagre		

N°	Food code	Food	Reference food code	Food synonyms
78	64-a	Pickles/Pepinillos picados, en vinagre		
79	66	Puerro picado crudo		
80	67	Puerro picado hervido		
81	68	Rabanito, crudo		
82	68-a	Rabanito en rodajas crudo		
83	68-b	Rabanito picado crudo		
84	70	Remolacha, cruda		
85	70-a	Remolacha en cubos cruda		
86	70-b	Remolacha rallada cruda		
87	71	Remolacha, hervida		
88	71-a	Remolacha en cubos hervida		
89	71-b	Remolacha rallada hervida		
90	73	Repollito de Bruselas, hervidos		
91	73-a	Repollito de Bruselas picados hervidos		
92	74	Repollo blanco picado crudo		
93	75	Repollo blanco hervido		
94	76	Rúcula, cruda		
95	77	Tomate perita crudo		
96	77-a	Tomate en rodajas crudo		
97	77-b	Tomate redondo crudo		
98	77-c	Tomate en cubos crudo		
99	78	Tomate, desecado		
100	79	Tomate, enlatado		
101	79-a	Tomate enlatado picado		
102	80	Tomate, hervido		Tomate escalfado
103	80-a	Tomate picado hervido	77-c	Tomate picado escalfado
104	81	Tomate, puré de tomate		
105	82	Tomate, puré de tomate, hervido	82	
106	83	Tomates cherry, crudos		
107	86	Zanahoria, cruda		
108	86-a	Zanahoria rallada cruda		
109	86-b	Zanahoria en listones cruda		
110	86-c	Zanahoria en cubos cruda		
111	87	Zanahoria, hervida		
112	87-a	Zanahoria, en cubos hervida		
113	87-b	Zanahoria rallada hervida		
114	88	Zapallito verde entero crudo		
115	89	Zapallito verde entero hervido		
116	89-a	Zapallito en rodajas hervido		
117	89-b	Zapallito en cubos hervido		
118	91	Zapallo, hervido		
119	91-a	Zapallo Anco en rodajas hervido		
120	91-b	Zapallo en cubos hervido		
121	92	Zucchini, crudo		Zapallito largo crudo - Calabacín crudo
122	92-a	Zucchini, crudo en rodajas		Zapallito largo crudo en rodajas - Calabacín crudo en rodajas
123	92-b	Zucchini, crudo rallado		Zapallito largo rallado - Calabacín rallado
124	92-c	Zucchini, crudo en listones		Zapallito largo en listones - Calabacín en listones
125	93	Zucchini, hervido		Zapallito largo hervido - Calabacín hervido
126	93-a	Zucchini picado hervido		Zapallito largo picado hervido - Calabacín picado hervido
127	94	Aceituna negra		
128	95	Aceituna verde		
129	95-a	Aceitunas descarozadas		
130	95-b	Aceitunas descarozadas picada		
131	97	Ananá		Piña
132	97-a	Ananá, rodaja		Piña en rodajas
133	97-b	Ananá picada		Piña picada
134	98	Ananá rodaja, enlatado (fruta y almíbar)		Piña enlatada
135	98-a	Ananá enlatado (fruta y almíbar) picado		Piña enlatado
136	99	Arándanos		
137	100	Banana		
138	100-a	Banana picada		
139	101	Cereza		
140	102	Ciruela		
141	102-a	Ciruela picada		
142	103	Ciruela pasa		Ciruela seca - Orejón de ciruela
143	104	Coco rallado		
144	106	Damasco		
145	106-a	Damasco picado		
146	107	Dátiles		
147	108	Durazno		
148	108-a	Durazno picado		
149	109	Durazno orejón		Durazno seco - Durazno deshidratado
150	110	Durazno, enlatado (fruta y almíbar)		
151	110-a	Durazno, enlatado (fruta y almíbar) mitad		

N°	Food code	Food	Reference food code	Food synonyms
152	110-b	Durazno, enlatado (fruta y almíbar) picado		
153	111	Durazno, enlatado light (fruta y almíbar)	110	
154	111-a	Durazno, enlatado light (fruta y almíbar) mitad	110-a	
155	111-b	Durazno, enlatado light (fruta y almíbar) picado	110-b	
156	112	Frambuesa		
157	113	Frutas abrillantadas		
158	114	Frutilla		Fresa
159	114-a	Frutilla picada		Fresa picada
160	117	Higo		
161	118	Higo desecado		Higo seco - Orejón de higo
162	121	Kiwi		
163	121-a	Kiwi picado		
164	122	Limón		
165	123	Mamón		Papaya
166	123-a	Mamón picado		Papaya picada
167	124	Mandarina		
168	124-a	Mandarina picada		
169	125	Mango		
170	125-a	Mango picado		
171	126	Manzana con piel		
172	126-a	Manzana con piel picada		
173	126-b	Manzana verde con piel		
174	127	Manzana sin piel		
175	127-a	Manzana sin piel picada		
176	129	Melón		
177	129-a	Melón, rodaja		
178	129-b	Melón picado		
179	131	Mora		
180	132	Naranja		
181	132-a	Naranja picada		
182	134	Palta del tipo chica		
183	134-a	Palta del tipo grande		
184	134-b	Palta en rodajas		
185	134-c	Palta en cubos		
186	134-d	Palta en pure		
187	136	Pera		
188	136-a	Pera picada		
189	137	Orejón de pera		Pera seca - Pera desecada
190	138	Pera, enlatada (fruta y almíbar)		
191	139	Pera, enlatada light (fruta y almíbar)	138	
192	140	Pomelo		
193	142	Sandía		
194	142-a	Sandía, rodaja		
195	142-b	Sandía picada		
196	143	Uva		
197	143-a	Uva racimo		
198	143-b	Uvas por separado		
199	144	Pasas de uva		
200	145	Almidón de maíz, crudo	686	Maicena
201	147	Amaranto, crudo		
202	151	Arroz blanco, hervido		
203	153	Arroz inflado azucarado		
204	155	Arroz integral, hervido		
205	157	Arroz yamaní, hervido	155	
206	159	Arveja, semilla seca entera / partida, hervida		
207	160	Avena, almohaditas		
208	161	Avena, cruda		Avena en hojuelas, cruda - Avena tradicional, cruda - Copos de avena, crudos - Avena arrollada, cruda
209	162	Avena, hervida		
210	163	Barra crocante de arroz		
211	164	Barra de cereal fortificada		Tipo Cereal Fort
212	165	Barra de cereal light		Tipo Flow Cereal
213	166	Barra de cereales, PROMEDIO		
214	166-a	Barra de cereal mix Chocolate & Almendras		Tipo Cereal mix
215	166-b	Barra de cereal mix Yoghurt & Frutilla		Tipo Cereal mix
216	166-c	Barra rellena de cereal mix Frutilla		Tipo Cereal mix
217	168	Batata, hervida		
218	168-a	Batatas en rodajas hervidas		
219	168-b	Batatas en cubos hervida		
220	169	Bizcochitos de arroz envasados (tipo galletita)		Tipo Bizcochos de arroz salados Capri Snacks
221	170	Bizcochitos de grasa envasados		Tipo bizcocho salado Don Satur
222	171	Bizcochitos de grasa envasados light		Tipo light bizcochos Don Satur
223	172	Bizcochos de grasa		Criollitos - Libritos - Pan con grasa - Bizcochitos cuadrados
224	173	Bizcochos tipo Bay Biscuit		
225	178	Brownie (panadería)		
226	179	Budín industrializado		
227	180	Budín industrializado con nueces, tipo navideño	179	

N°	Food code	Food	Reference food code	Food synonyms
228	184	Capelettis frescos, artesanal, hervido		
229	185	Cereal desayuno, arítos frutales		Cereal de aros frutales
230	186	Cereal desayuno, aritos sabor miel		Cereal de aros sabor a miel
231	187	Cereal desayuno, bolitas de chocolate		Choco bolitas
232	189	Cereal desayuno, copos azucarados, sin fortificar		Copos de maíz azucarados
233	188	Cereal desayuno, copos azucarados, fortificados	189	Copos de maíz azucarados fortificados
234	191	Cereal desayuno, copos de maíz sin azúcar, sin fortificar		
235	192	Cereal desayuno, salvado de trigo tipo bastoncitos		
236	194	Cheesecake (panadería / confitería)		
237	195	Chipá		
238	196	Chipá preparado a partir de polvo premezcla	195	
239	197	Chipaco o pan con chicharrón		
240	199	Choclo, enlatado cremoso		
241	200	Choclo, enlatado en grano		
242	201	Choclo, hervido entero (peso desgranado)		
243	201-a	Choclo fresco, picado hervido (peso desgranado)		
244	201-b	Choclo fresco, desgranado hervido		
245	202	Churros		
246	203	Coquitos (panadería)		
247	206	Cucurucho / cubanito		
248	208	Facturas rellenas		
249	208-a	Vigilante		
250	209	Facturas simples		
251	209-a	Facturas simples (Medialuna salada)		
252	209-b	Facturas simples (Medialuna dulce)		
253	213	Fideos frescos, al huevo, hervidos		
254	218	Fideos secos, fortificados, hervidos		
255	219	Fideos secos, hervidos	218	
256	223	Galleta marinera		
257	224	Galleta marinera con semillas / galletitas crackers con semillas		
258	225	Galleta marinera integral		
259	226	Galleta marinera, light en grasas		
260	235	Galletitas de agua, promedio		
261	235-a	Bizcochito salado tipo 9 de Oro		
262	235-b	Bizcochito salado tipo Don Satur		
263	235-c	Galletita de agua tipo Granix sándwich		
264	236	Galletitas de salvado o integrales		
265	236-a	Galletita integral tipo Granix sándwich		
266	236-b	Galletita integral tipo Cereal Granix		
267	237	Galletitas dulces con centro de membrillo o similar (tipo Pepas)		
268	238	Galletitas dulces con chips de chocolate		
269	242	Galletitas dulces rellenas		
270	242-a	Galletitas tipo Oreo		
271	243	Galletitas dulces rellenas bañadas en chocolate		
272	244	Galletitas dulces simples		
273	244-a	Galletita dulce integral tipo Granovita Algarroba		
274	244-b	Bizcochos agridulces tipo 9 de Oro		
275	244-c	Porteniñitas, Bagley		
276	244-d	Bizcocho dulce Don Satur		
277	245	Galletitas dulces integrales tipo Frutigran		
278	252	Garbanzos, hervidos		
279	251	Garbanzos, enlatados	252	
280	254	Granola		
281	255	Grisines		
282	256	Grisines sin sal	255	
283	262	Harina de chía		
284	265	Harina de maíz, hervida		Polenta
285	270	Harina de trigo integral, cruda		
286	271	Harina de trigo, cruda		
287	275	Lentejas, hervidas		
288	274	Lentejas, enlatadas	275	
289	278	Madalenas		Muffins
290	279	Madalenas fortificadas	278	
291	280	Madalenas rellenas	278	
292	282	Maíz, grano entero, hervido	201-b	
293	293	Milanesa de soja, frita		
294	295	Ñoquis de papa frescos, artesanales, hervidos		
295	297	Ñoquis de papa, envasados, hervidos	295	
296	298	Ñoquis preparados a partir de polvo premezcla	295	
297	299	Ñoquis preparados a partir de polvo premezcla, sin tacc	295	
298	301	Pan blanco, tipo molde, lacteado		
299	301-a	Pan lactál tipo molde (rodaja)		
300	301-b	Pan lactal tipo molde (triángulo)		
301	308	Pan de miga		
302	308-a	Pan de miga triángulo		
303	309	Pan de molde con salvado		
304	309-a	Pan de molde con salvado (rodaja)		

N°	Food code	Food	Reference food code	Food synonyms
305	309-b	Pan de molde con salvado (triángulo)		
306	310	Pan de molde con salvado sin sal	309	
307	310-a	Pan de molde con salvado sin sal (rodaja)	309-a	
308	310-b	Pan de molde con salvado sin sal (triángulo)	309-b	
309	311	Pan de salvado		
310	311-a	Pan de salvado miñón/francés/Felipe		
311	311-b	Pan de salvado, rodaja miñón/francés/felipe		
312	312	Pan de salvado con semillas		
313	313	Pan de salvado sin sal (panadería)	311	
314	315	Pan dulce		
315	317	Pan francés o pan casero sin agregado de grasa o aceite		
316	317-a	Pan francés o pan casero, rodaja		
317	318	Pan francés sin sal	317	
318	318-a	Pan francés sin sal, rodaja	317-a	
319	321	Pan para panchos o hamburguesas, envasado		
320	321-a	Pan para hamburguesas		
321	321-b	Pan para panchos		
322	323	Pan rallado		
323	324	Pan rallado, fortificado	323	
324	325	Pan rallado, sin tacc	323	
325	329	Papa, hervida		
326	329-a	Papa en rodajas hervida		
327	329-b	Papa en cubos hervida		
328	330	Papa, puré instantáneo (polvo)		
329	336	Pochoclo acaramelado		
330	337	Pochoclo salado		
331	341	Porotos, enlatados		
332	342	Porotos, hervidos		
333	342-a	Porotos rojos hervidos		
334	342-b	Porotos blancos hervidos		
335	342-c	Porotos negros hervidos		
336	345	Puflito		
337	346	Quinoa inflada		
338	348	Quinoa, semilla, hervida		
339	351	Ravioles frescos, artesanales, hervidos		
340	351-a	Sorrentinos frescos, artesanales, hervidos		
341	358	Salvado de avena		
342	359	Salvado de trigo		
343	362	Sémola, cruda		
344	360	Sémola fortificada, cruda	362	
345	366	Soja, porotos, hervidos		
346	367	Sushi, promedio		
347	370	Tofu		Queso vegano de soja
348	370-a	Tofu feta		Queso vegano de soja feta
349	370-b	Tofu picado		Queso vegano de soja picado
350	374	Torta frita		
351	376	Tortillas de trigo, envasadas		Tortillas para taco
352	378	Tostadas de gluten		
353	379	Tostadas de mesa	378	
354	380	Tostadas integrales	378	
355	381	Tostadas light	378	
356	382	Tostadas sin sal	378	
357	383	Tostaditas saladas, saborizadas, tipo snacks - Twistos		
358	386	Trigo inflado azucarado		
359	389	Turrón de maní con oblea tipo golosina		
360	390	Turrón tipo navideño		
361	391	Tutuca		
362	392	Alimento a base de leche entera y café, fortificado con calcio, varios sabores, listo para consumir		
363	393	Bebida láctea parcialmente descremada fluida, baja en lactosa, fortificada con vitaminas A, D, B2, B9 y zinc		
364	394	Flan envasado listo para consumir		
365	395	Flan envasado listo para consumir light		
366	396	Leche condensada		
367	400	Leche de vaca, ordeñada (sin ningún tipo de procesamiento)		Leche fresca de vaca
368	401	Leche descremada en polvo, fortificada con vitaminas A y D		
369	403	Leche descremada fluida, con 50% más de proteínas		
370	406	Leche entera en polvo, deslactosada, fortificada con vitaminas A y D *	401	
371	407	Leche entera en polvo, fortificada con hierro	401	
372	408	Leche entera en polvo, fortificada con vitaminas A y D	401	
373	412	Leche entera en polvo, sin fortificación	401	Leche en polvo - Leche de vaca en polvo
374	413	Leche entera fluida, fortificada con hierro		
375	414	Leche entera fluida, con azúcar, lista para consumir		
376	415	Leche entera fluida, con CLA, fortificada con vitaminas A y D		
377	416	Leche entera fluida, con vitaminas A y D		

N°	Food code	Food	Reference food code	Food synonyms
378	417	Leche entera fluida, deslactosada, fortificada con vitaminas A y D		
379	418	Leche entera fluida, fortificada con vitaminas A, D y B9		
380	419	Leche entera fluida, fortificada con vitaminas A, D, C, B9 y extra calcio		
381	420	Leche entera fluida, sin fortificación		Leche líquida - Leche de vaca líquida
382	442	Leche parcialmente descremada fluida, con fitoesteroles y ácidos Grasos omega 3, fortificada con calcio y vitaminas A, D y E		
383	443	Leche parcialmente descremada fluida, deslactosada, fortificada con vitaminas A y D		
384	444	Leche parcialmente descremada fluida, fortificada con hierro		
385	445	Leche parcialmente descremada fluida, fortificada con vitaminas A y D		
386	446	Leche parcialmente descremada fluida, fortificada con vitaminas A, D, C y ácido fólico		
387	447	Leche parcialmente descremada fluida, sin fortificación		
388	448	Leche parcialmente descremada, sabor chocolate o dulce de leche, fortificada con vitaminas A y D, lista para consumir		
389	450	Leche semidescremada en polvo, deslactosada	401	
390	453	Postre de leche listo para consumir tipo Danonino		
391	454	Postre de leche listo para consumir tipo Serenito		
392	455	Yogur descremados con probióticos		
393	456	Yogur entero con probióticos		
394	457	Yogur con fitoesteroles		
395	458	Yogur descremado		
396	459	Yogur descremado bebible		
397	460	Yogur descremado con cereales		
398	461	Yogur descremado con frutas		
399	462	Yogur descremado con frutas y cereales		
400	463	Yogur descremado fortificado con calcio		
401	464	Yogur entero bebible saborizado		
402	465	Yogur entero con cereales		
403	466	Yogur entero con frutas		
404	467	Yogur entero con frutas y cereales		
405	468	Yogur entero natural		
406	469	Yogur entero saborizado		
407	470	Yogur fortificado con hierro		
408	473	Queso Azul		
409	478	Queso crema entero, untable		
410	474	Queso blanco descremado, untable	478	
411	475	Queso blanco semidescremado, untable	478	
412	476	Queso Cheddar		
413	479	Queso Cremoso		
414	480	Queso Cuartirolo	479	
415	490	Queso Holanda		
416	490-a	Queso Holanda en fetas		
417	490-b	Queso Holanda en cubos		
418	491	Queso Mar del Plata		
419	491-a	Queso Mar del Plata en fetas	490-a	
420	491-b	Queso Mar del Plata en cubos	490-b	
421	472	Queso Pategrás		
422	472-a	Queso Pategrás en fetas	490-a	
423	472-b	Queso Pategrás en cubos	490-b	
424	485	Queso Fontina		
425	485-a	Queso fontina en fetas	490-a	
426	485-b	Queso fontina en cubos	490-b	
427	489	Queso Gruyére		
428	489-a	Queso gougérette en fetas	490-a	
429	489-b	Queso gougé en cubos	490-b	
430	492	Queso mascarpone	478	
431	493	Queso mozzarella		
432	493-a	Queso mozzarella rallado	505-a	
433	499	Queso pasta semidura, promedio		
434	499-a	Queso en barra, feta		
435	500	Queso port salut sin sal light	479	
436	501	Queso provolone		
437	502	Queso rallado envasado		
438	494	Queso parmesano	502	
439	503	Queso reggianito	502	
440	504	Queso roquefort	473	
441	505	Queso sardo		
442	505-a	Queso sardo rallado		
443	505-b	Queso sardo en cubos		
444	506	Queso sbrinz	502	
445	507	Queso untable tipo Finlandia	478	

N°	Food code	Food	Reference food code	Food synonyms
446	508	Queso untable tipo Finlandia light	478	
447	509	Queso untable tipo Tholem	478	
448	510	Queso untable tipo Tholem light	478	
449	513	Ricota		
450	514	Ricota descremada	513	
451	519	Cantimpalo (fiambre)		Salame Cantimpalo - Chorizo Cantimpalo
452	522	Cerdo, promedio horno/parrilla		
453	522-a	Paleta de cerdo asada		
454	524	Chinchulines, tripa gorda horno/parrilla		
455	528	Chorizo horno/parrilla		
456	538	Hamburguesa de carne vacuna cocida, industrializada		
457	537	Hamburguesa de carne vacuna cocida	538	
458	539	Hamburguesa de carne vacuna cocida, industrializada light	538	
459	540	Hamburguesa de carne vacuna cocida, industrializada, rebozada	538	
460	542	Hamburguesa de pollo cocida, industrializada		
461	541	Hamburguesa de pollo cocida	542	
462	544	Hígado rehogado/plancha		
463	548	Jamón cocido		
464	549	Jamón crudo		
465	552	Lengua, hervida		
466	553	Lomito ahumado		
467	554	Longaniza		
468	555	Matambre (fiambre)		
469	556	Milanesa de carne vacuna rebozada frita		
470	557	Milanesa de pollo rebozada frita		
471	560	Mollejas, a la parrilla		
472	562	Mondongo, hervido		
473	563	Morcilla		
474	564	Mortadela		
475	568	Paleta (fiambre)	548	
476	569	Panceta frita		
477	569-a	Panceta frita picada en tiras		
478	569-b	Panceta frita picada en cubos		
479	570	Pastrón		
480	571	Paté / picadillo		
481	577	Pollo con piel horno/parrilla		
482	577-a	Carne de pollo - Patamuslo con piel horno/parrilla		
483	577-b	Carne de pollo - Pata con piel horno/parrilla		
484	577-c	Carne de pollo - Muslo con piel horno/parrilla		
485	577-d	Carne de pollo - Ala con piel horno/parrilla		
486	579	Pollo sin piel horno/parrilla		
487	579-a	Carne de pollo - Patamuslo sin piel horno/parrilla		
488	579-b	Carne de pollo - Pata sin piel horno/parrilla		
489	579-c	Carne de pollo - Muslo sin piel horno/parrilla		
490	579-d	Carne de pollo - Ala sin piel horno/parrilla		
491	583	Pollo, pechuga sin piel horno/parrilla		
492	586	Riñón horno/parrilla		
493	587	Salame		
494	588	Salchicha de viena		
495	588-a	Salchicha de viena ahumada		
496	589	Salchicha de viena light	588	
497	589-a	Salchicha de viena light ahumada	588-a	
498	591	Salchicha parrillera, a la parrilla	528	
499	597	Vacuno, cortes grasos, promedio, hervido		
500	597-a	Carne vacuna - Alita hervida		
501	597-b	Carne vacuna - Vacío hervido		
502	597-c	Carne vacuna - Falda hervido		
503	597-d	Carne vacuna - Carne picada común hervida		
504	598	Vacuno, cortes grasos, promedio, horno/parrilla		
505	598-a	Carne vacuna - Matambre horno/parrilla		
506	598-b	Carne vacuna - Aguja horno/parrilla		
507	598-c	Carne vacuna - Vacío horno/parrilla		
508	598-d	Carne vacuna - Asado horno/parrilla		
509	598-e	Carne vacuna - Albóndiga		
510	599	Vacuno, cortes magros, promedio, crudo		
511	600	Vacuno, cortes magros, promedio, hervido		
512	601	Vacuno, cortes magros, promedio, horno/parrilla		
513	601-a	Carne vacuna - Filet cuadrada horno/parrilla		
514	601-b	Carne vacuna - Peceto horno/parrilla		
515	601-d	Carne vacuna - Bola de lomo horno/parrilla		
516	601-e	Carne vacuna - Cuadrada horno/parrilla		
517	601-f	Carne vacuna - Nalga horno/parrilla		
518	603	Vacuno, cortes semigrasos, promedio, hervido		
519	603-a	Carne vacuna - Palomita hervida		
520	603-b	Carne vacuna - Carne picada especial hervida	597-d	
521	604	Vacuno, cortes semigrasos, (ej: lomo, carne picada especial, roast beef, paleta, bife angosto, tortuguita, palomita), promedio, horno/parrilla		

N°	Food code	Food	Reference food code	Food synonyms
522	604-a	Carne vacuna - bife ancho sin hueso horno/parrilla		
523	604-b	Carne vacuna - Osobuco horno/parrilla		
524	604-c	Carne vacuna - Paleta horno/parrilla		
525	604-d	Carne vacuna - Lomo horno/parrilla		
526	609	Huevo de gallina, clara, hervida		
527	611	Huevo de gallina, entero, hervido		Huevo duro
528	611-a	Huevo de gallina, medio, hervido		Medio huevo duro
529	613	Huevo de gallina, yema, hervida		
530	616	Anchoa, enlatada en aceite		
531	617	Atún, enlatado al natural		
532	618	Atún, enlatado en aceite	617	
533	622	Caballa, enlatada		
534	635	Merluza		
535	635-a	Filet de pescado de mar, merluza a la plancha		
536	652	Sardinas, enlatadas al natural		
537	653	Sardinas, enlatadas en aceite	652	
538	656	Aceite comestible mezcla		
539	657	Aceite de canola		
540	658	Aceite de chía		
541	659	Aceite de coco		
542	660	Aceite de girasol		
543	661	Aceite de girasol alto oleico		
544	662	Aceite de maíz		
545	663	Aceite de oliva		
546	664	Aceite de sésamo		
547	665	Aceite de soja		
548	666	Aceite de uva		
549	667	Aceite mezcla con oliva		
550	668	Rocío vegetal		
551	669	Leche de almendras		
552	670	Almendra		
553	670-a	Almendra picada		
554	671	Avellana		
555	671-a	Avellana picada		
556	672	Maní tostado salado		
557	673	Maní tostado sin sal	672	
558	674	Manteca de maní		Mantequilla de maní
559	676	Nuez, una mariposa (mitad de una nuez)		
560	676-a	Nuez picada		
561	677	Pistacho salado		
562	677-a	Pistacho picado salado		
563	678	Semilla de amapola		
564	679	Semilla de chía		
565	680	Semilla de girasol		
566	681	Semilla de girasol con sal y con cáscara		
567	682	Semilla de lino		
568	683	Semilla de sésamo		
569	684	Semilla de zapallo		
570	685	Azúcar blanca molida		
571	686	Azúcar impalpable		
572	687	Azúcar morena / azúcar mascabo		
573	688	Dulce de batata		
574	689	Dulce de batata light	688	
575	690	Dulce de leche		
576	691	Dulce de leche light	690	
577	692	Dulce de membrillo		
578	693	Dulce de membrillo light	692	
579	699	Miel		
580	694	Glucosa	699	
581	695	Jalea tipo membrillo	699	
582	696	Kero o melaza	699	
583	697	Mermelada de frutas		
584	698	Mermelada de frutas light	697	
585	700	Alfajor de arroz		
586	701	Alfajor de chocolate		
587	702	Alfajor de chocolate con relleno tipo cofler mousse		
588	704	Alfajor de fruta		
589	705	Alfajor de maicena		
590	707	Alfajor light		
591	708	Bananita con cobertura de chocolate		
592	709	Baño de repostería para cubrir tortas		
593	710	Barra de arroz rellena bañada		
594	711	Barra de chocolate		Tipo Kinder chocolate
595	712	Bocadito con dulce de leche		Tipo bocadito Cabsha
596	713	Bombón		Tipo Bon O Bon
597	714	Bombones de fruta		
598	715	Cacao amargo		
599	716	Cacao en polvo sin fortificar	715	
600	717	Cacao en polvo	715	Tipo Nesquick

N°	Food code	Food	Reference food code	Food synonyms
601	718	Caramelos duros		
602	719	Caramelos duros light	718	Tipo caramelos clight sin azúcar
603	720	Caramelos masticables		Tipo (Billiken, Sugus, Arcor fruta...)
604	721	Caramelos rellenos		Tipo (De miel, Frutales, Con dulce de leche…)
605	723	Chicles sin azúcar		Tipo (TopLine, Beldent…)
606	722	Chicles	723	
607	724	Chocolate 60% cacao o más		Tipo Aguila
608	724-a	Chocolate 80% cacao		Tipo Aguila
609	725	Chocolate amargo	724-a	
610	726	Chocolate blanco		Tipo chocolatín Arcor
611	727	Chocolate con almendras		Tipo Milka
612	728	Chocolate con maní		Tipo Cofler Block
613	729	Chocolate light		
614	730	Chocolate para taza		
615	731	Chocolatín		Tipo Chocolatín Arcor leche
616	731-a	Chocolate con leche		Tipo Milka leche
617	732	Chupetín		
618	732-a	Chupetín tipo Pico dulce		
619	732-b	Chupetín tipo Chupetóncito		
620	732-c	Chupetín tipo Wamis		
621	733	Conito de dulce de leche		
622	734	Cubanito relleno		
623	735	Garrapiñada de maní		
624	736	Gomitas de gelatina		
625	737	Huevo de chocolate		Tipo Kinder
626	738	Lentejas de chocolate		Tipo Rocklets
627	739	Maní con chocolate		Maní cubierto con chocolate - Maní con cobertura de chocolate
628	740	Medallón de menta y chocolate		
629	741	Merengues / merenguitos		
630	742	Alfajor minitorta bañada		
631	743	Oblea bañada tipo Kit Kat		
632	744	Obleas bañadas tipo Nugaton		
633	744-a	Obleas bañadas tipo Bon o Bon		
634	745	Pasas de uva con chocolate	739	Pasas cubiertas con chocolate - Pasas con cobertura de chocolate
635	746	Pastillas tipo Tic Tac		
636	747	Pastillas sin azúcar tipo Tic Tac	746	
637	748	Postre a base de pasta de maní, tipo Mantecol		
638	749	Rhodesia		
639	750	Tableta de dulce de leche		
640	751	Tableta de dulce de leche light		
641	752	Tita		
642	753	Crema chantilly (lista para consumir, aerosol o industrial preparada)		
643	754	Crema de leche		
644	755	Crema light	754	
645	756	Grasa de cerdo		
646	757	Grasa de pollo	756	
647	758	Grasa vacuna	756	
648	759	Manteca		
649	760	Manteca light	759	
650	761	Margarina	759	
651	762	Margarina untable light	759	
652	764	Maní japonés		Maní saborizado
653	765	Nachos		Tipo Doritos
654	766	Palitos aireados de harina de maíz o de colores tipo Chizitos		
655	767	Palitos salados		
656	768	Papas fritas de copetín		
657	769	Snacks saborizados salados a base de maíz tipo Conitos		
658	770	Snacks saborizados salados a base de sémola de arroz, horneados, tipo Palitos de arroz / aros de cebolla		
659	771	Aceto balsámico		
660	774	Jugo de limón, envasado		
661	775	Ketchup		
662	776	Mayonesa		
663	777	Mayonesa de soja	776	
664	778	Mayonesa light	776	
665	779	Mostaza		
666	784	Salsa chimichurri envasada		
667	785	Salsa de soja		
668	787	Salsa Golf		
669	788	Vinagre		
670	789	Caldos en cubo		
671	790	Caldos en polvo		
672	791	Caldos en polvo light		
673	792	Caldos en polvo sin sal light		
674	792-a	Caldos en cubo sin sal light		

N°	Food code	Food	Reference food code	Food synonyms
675	793	Saborizador en cubos		
676	794	Saborizador en polvo		
677	796	Sopa crema instantánea, polvo para preparar (sabores varios)		
678	797	Sopa crema instantánea, polvo para preparar light (sabores varios)		
679	800	Sopa de fideos deshidratada envasada para preparar		Ramen
680	801	Sopa de vegetales instantánea, polvo para preparar		
681	802	Arroz con leche envasado listo para consumir		
682	803	Bombón helado		
683	804	Gelatina envasada lista para consumir		
684	806	Gelatina preparada a partir de polvo		
685	805	Gelatina light, preparada a partir de polvo	806	
686	807	Helado de agua (heladería)		
687	808	Helado de agua (palito)		
688	809	Helado de crema (heladería)		
689	810	Helado de crema envasado		
690	811	Helado envasado con cucurucho		
691	815	Sal		
692	816	Sal dietética o modificada	815	
693	817	Sal dietética, sin sodio	815	
694	818	Sal Marina		
695	819	Sal modificada, 33% Cloruro de sodio	818	
696	820	Sal modificada, 66% Cloruro de sodio	818	
697	821	Agua saborizada Aquarius, varios sabores		
698	822	Agua saborizada Awafrut, varios sabores		
699	823	Agua saborizada con azúcar		
700	824	Agua saborizada Levité, varios sabores		
701	825	Agua saborizada tipo Sierra de los Padres, Manaos, otros		
702	826	Amargo serrano		
703	827	Leche de soja, sabor natural		
704	828	Bebida a base de soja y jugo, varios sabores		
705	829	Bebida a base de soja y jugo, varios sabores light		
706	830	Gaseosa con azúcar		
707	831	Gatorade, varios sabores		
708	832	Jugo concentrado con azúcar, para diluir		
709	834	Jugo de fruta envasado, varios sabores		
710	836	Powerade, varios sabores		
711	837	Agua saborizada Flip light		
712	838	Agua saborizada H2O, varios sabores		
713	839	Agua saborizada Levité Cero, varios sabores light		
714	840	Agua saborizada light		
715	841	Agua saborizada We o Ser ligth		
716	842	Amargo serrano light		
717	843	Gaseosa light		
718	844	Gatorade Zero, varios sabores (sin azúcar)		
719	845	Jugo en polvo BC		
720	846	Jugo en polvo light		
721	847	Jugo tipo BC, varios sabores (alimento líquido dietético con 50% de jugo y pulpa)		
722	848	Powerade Zero, varios sabores (sin azúcar)		
723	849	Ananá Fizz		
724	850	Aperitivo, tipo Gancia		
725	851	Bebida destilada		
726	852	Bebida energizante		
727	853	Cerveza con alcohol		
728	854	Cerveza sin alcohol		
729	855	Champagne		
730	856	Chicha, bebida tradicional obtenida por fermentación del maíz		
731	857	Dr. Lemon		
732	858	Fernet		
733	859	Licor		
734	860	Sidra		
735	861	Vermouth		
736	862	Vino blanco		
737	863	Vino espumante, tipo Frizze		
738	864	Vino Mistela		
739	865	Vino tinto		
740	866	Vodka		
741	867	Jugo de arándanos, natural		
742	868	Jugo de fruta, exprimido sin azúcar agregada, envasado		
743	869	Jugo de fruta, pasteurizado no reconstituido		
744	870	Jugo de limón, exprimido		
745	871	Jugo de naranja, exprimido		
746	872	Jugo de papaya, natural		
747	873	Jugo de pomelo, exprimido		
748	874	Café instantáneo (polvo o granulado para preparar)		
749	875	Café instantáneo descafeinado (polvo o granulado para preparar)	874	

N°	Food code	Food	Reference food code	Food synonyms
750	876	Café preparado a partir de grano molido (tipo café de filtro)		
751	877	Capucchino preparado		
752	878	Malta instantánea en polvo		
753	879	McDonald's, BigMc		
754	880	McDonald's, Cajita Feliz, Hamburguesa con queso Grill		
755	881	McDonald's, Cajita Feliz, Hamburguesa Grill		
756	883	McDonald's, Cajita Feliz, McNuggets X4		
757	884	McDonald's, Cajita Feliz, Papas Kids		
758	885	McDonald's, Cajita Feliz, Papas pequeñas		
759	887	McDonald's, Club House		
760	888	McDonald's, Clubhouse/ Guacamole/ Cryspi Onion BBQ con pollo crispy		
761	889	McDonald's, Clubhouse/ Guacamole/ Cryspi Onion BBQ con pollo grill	888	
762	890	McDonald's, Cono		
763	891	McDonald's, Cuarto de Libra		
764	892	McDonald's, Doble Cuarto de Libra con queso		
765	893	McDonald's, Hamburguesa	881	
766	894	McDonald's, Hamburguesa con queso	880	
767	895	McDonald's, Mc Flurry Oreo		
768	896	McDonald's, Mcnífica		
769	897	McDonald's, McNuggets x 10		
770	898	McDonald's, McPollo Grill		
771	899	McDonald's, Papas grandes		
772	900	McDonald's, Papas medianas		
773	901	McDonald's, Papas pequeñas	885	
774	903	McDonald's, Sundae chocolate		
775	904	McDonald's, Sundae dulce de leche		
776	905	McDonald's, Sundae frutilla		
777	906	McDonald's, Triple Mac		
778	907	Dextrosa en polvo (gramo)	685	
779	908	Ensure Plus (mililitro)		
780	909	Ensure Plus drink (mililitro)		
781	912	Fortisip Standard (mililitro)		
782	913	Frebini energy drink (mililitro)		
783	915	Jevity (mililitro)		
784	917	MCT Oil-triglicéridos de cadena media (Nutricia Bagó) (mililitro)		
785	920	Osmolite NH (mililitro)		
786	923	Supportan (mililitro)		
787	927	Supportan drink (mililitro)		
788	931	Mix de semillas		
789	932	Semillas de girasol tostada		
790	933	Semillas de sésamo tostada		
791	934	Brotes de alfalfa		
792	935	Pan tipo molde blanco tostado		
793	935-a	Pan tipo molde blanco tostado (rodaja)		
794	935-b	Pan tipo molde blanco tostado (triángulo)		
795	936	Manzana sin piel hervida		
796	937	Ensalada de frutas (kiwi, mandarina, manzana)		
797	938	Maní crudo		
798	939	Tofu frito		Queso vegano de soja frito
799	939-a	Tofu frito feta		Queso vegano de soja frito en feta
800	939-b	Tofu frito picado		Queso vegano de soja frito picado
801	940	Curry		
802	941	Orégano seco		
803	942	Cúrcuma		
804	943	Canela		
805	944	Pimienta negra		
806	946	Batata horneada con aceite y sal		
807	946-a	Batata en rodajas horneada con aceite y sal		
808	946-b	Batata en cubos horneada con aceite y sal		
809	947	Ensalada de tomate, lechuga y cebolla		
810	948	Ensalada de zanahoria, lechuga y tomate		
811	949	Repollo blanco picado salteado con aceite y sal		
812	950	Repollo colorado picado salteado con aceite y sal		
813	951	Papa bastones al horno con aceite y sal		
814	952	Manzana sin piel picada cocida en microondas		
815	953	Repollo colorado picado crudo		
816	954	Zanahoria salteada con aceite y sal		
817	954-a	Zanahoria rallada salteada con aceite y sal		
818	954-b	Zanahoria en cubos salteada con aceite y sal		
819	955	Zapallo horneado		
820	955-a	Zapallo Anco en rodajas horneado		
821	955-b	Zapallo en cubos horneado		
822	956	Zapallo salteado con aceite y sal		
823	956-a	Zapallo Anco en rodajas salteado con aceite y sal		
824	956-b	Zapallo en cubos salteado con aceite y sal		
825	957	Remolacha salteada con aceite y sal		
826	957-a	Remolacha en cubos salteada con aceite y sal		
827	957-b	Remolacha rallada salteada con aceite y sal		

N°	Food code	Food	Reference food code	Food synonyms
828	958	Acelga salteada con aceite y sal	4	
829	959	Brócoli salteado con aceite y sal		
830	959-a	Brócoli arbolito salteado con aceite y sal		
831	960	Coliflor arbolito salteado con sal y aceite		
832	960-a	Coliflor picado salteado con sal y aceite		
833	961	Morrón rojo picado hervido		
834	962	Morrón verde picado hervido		
835	962-a	Morrón amarillo picado hervido		
836	963	Repollo colorado hervido		
837	964	Repollito de bruselas salteado con aceite y sal		
838	964-a	Repollito de bruselas picado salteado con aceite y sal		
839	965	Ciboulette picado		
840	966	Espinaca hoja salteada con sal y aceite		
841	966-a	Espinaca picada salteada con sal y aceite		
842	967	Champiñones picados hervidos		
843	968	Champiñones picados salteados		
844	969	Champiñones en lata salteados		
845	970	Tomate cortado en cubos salteado con aceite y sal		
846	971	Puerro picado salteado con sal y aceite		
847	972	Berenjena en rodajas salteada con sal y aceite		
848	972-a	Berenjena en cubos salteada con sal y aceite		
849	973	Cebolla hervida		
850	973-a	Cebolla picada en cubos hervida		
851	974	Ajo hervido		
852	975	Papa frita con sal		
853	975-a	Papas fritas con sal en bastones		
854	975-b	Papas fritas con sal en rodajas		
855	976	Batatas fritas con sal		
856	976-a	Batatas fritas con sal en rodajas		
857	976-b	Batatas fritas con sal en cubos		
858	977	Achicoria, hoja, salteada con sal y aceite		
859	977-a	Achicoria picada salteada con sal y aceite		
860	978	Espárrago salteado con sal y aceite		
861	978-a	Espárrago picado salteado con sal y aceite		
862	979	Huevo frito con sal		
863	980	Huevo hervido tipo poché		
864	981	Huevo horneado con aceite y sal		
865	979-a	Huevo batido frito con sal		
866	983	Clara de huevo salteada con sal y aceite		
867	984	Yema de huevo salteada con aceite y sal		
868	985	Castañas de cajú tostadas		
869	985-a	Castañas de cajú picada tostadas		
870	986	Almendras picadas tostadas		
871	987	Avellana picada tostada		
872	988	Banana deshidratada		
873	989	Mandioca salteada con aceite y sal		
874	990	Palmitos picados salteados con aceite y sal		
875	994	Arvejas frescas enlatadas salteadas con aceite		
876	995	Pan de miga integral		
877	995-a	Pan de miga integral triángulo		
878	996	Castañas de cajú		
879	997	Pistacho picado tostado		
880	998	Arroz integral salteado con aceite y sal		
881	1000	Arvejas frescas salteadas con aceite y sal		
882	1001	Romero		
883	1007	Snaks - Rex		
884	1008	Snaks - Saladix		
885	1009	Tomillo seco		
886	1001-a	Romero seco		
887	1011	Albahaca seca		
888	1012	Comino		
889	1013	Nuez moscada		
890	1014	Cebolla deshidratada		
891	1015	Carne de pollo - Ala o pechuga hervida desmenuzada		
892	1016	Carne de pollo - Pechuga media presa sin piel hervida		
893	1017	Carne de pollo - Pechuga media presa sin piel frita		
894	1002	Carne de pollo - Patamuslo sin piel hervido		
895	1003	Carne de pollo - Pata sin piel hervido		
896	1004	Carne de pollo - Muslo sin piel hervido		
897	1005	Carne de pollo - Ala sin piel hervida		
898	1049	Carne de pollo - Patamuslo con piel hervido		
899	1050	Carne de pollo - Pata con piel hervido		
900	1051	Carne de pollo - Muslo con piel hervido		
901	1052	Carne de pollo - Ala con piel hervida		
902	1053	Carne de pollo - Patamuslo sin piel frito		
903	1054	Carne de pollo - Pata sin piel frito		
904	1055	Carne de pollo - Muslo sin piel frito		
905	1056	Carne de pollo - Ala sin piel frita		
906	1057	Carne de pollo - Patamuslo con piel frito		
907	1058	Carne de pollo - Pata con piel frito		
908	1059	Carne de pollo - Muslo con piel frito		

N°	Food code	Food	Reference food code	Food synonyms
909	1060	Carne de pollo - Ala con piel frita		
910	527-a	Carne vacuna - Chorizo horno/parrilla		
911	1023	Carne de pollo - Nuggets o patitas de pollo fritos		
912	1023-a	Carne de pollo - Medallón frito		
913	1027	Pelón		
914	1027-a	Pelón picado		
915	1028	Tortillas de trigo integral, envasadas		
916	1029	Levadura Nutricional (Suplemento)		
917	1030	Arepa de harina de maíz a la plancha		
918	1031	Tomate en lata salteado con aceite		
919	1032	Avena hidratada		
920	1033	Soja texturizada hidratada		
921	1034	Menta fresca		
922	1034-a	Menta fresca picada		
923	1036	Carne de pescado - Filet de Río cocido		
924	1036-a	Filet de pescados de río, Tararira a la plancha		
925	1037	Carne de cerdo - Costeleta a la sartén		
926	1038	Carne de cerdo - Costeleta a la olla		
927	1039	Carne de cerdo - Costeleta asada		
928	1040	Carne de cerdo - Matambre (corte de carne) asado		
929	1041	Carne de cerdo - Tira de costillas asada		
930	1044	Extracto doble de tomate		
931	1045	Carne de pollo - Pechuga filet sin piel a la olla		
932	1046	Carne de pollo - Pechuga filet sin piel asada		
933	1047	Carne de pollo - Pechuga filet sin piel frita		
934	1048	Alfajor triple (promedio de marcas)		
935	1048-a	Alfajor triple relleno con dulce de leche y maní tipo Block		
936	1048-b	Alfajor triple relleno con dulce de leche tipo Tofi		
937	1061	Empanada con relleno de carne		
938	1062	Empanada con relleno de soja texturizada	1061	
939	1063	Empanada con relleno de soja texturizada 100% de origen vegetal	1061	
940	1064	Empanada con relleno de verdura sin huevo	1061	
941	1065	Empanada con relleno de verdura con huevo	1061	
942	1066	Empanada con relleno de verdura y ricota	1061	
943	1067	Empanada con relleno de choclo y huevo	1061	
944	1068	Empanada con relleno de choclo y salsa blanca	1061	
945	1069	Empanada con relleno de verdura 100% de origen vegetal	1061	
946	1070	Empanada con relleno de cebolla y queso	1061	
947	1071	Empanada con relleno de queso y huevo	1061	
948	1072	Sopa de verduras		
949	1073	Sopa de verduras y carne		
950	1074	Sopa de verduras y cereales (Arroz y Fideos)		
951	1075	Sopa de verduras y cereales (Avena y Sémola)		
952	1076	Sopa de cereales		
953	1077	Guiso con cereales		
954	1078	Guiso con legumbres		
955	1079	Guiso con carne y cereales		
956	1080	Guiso con cereales y legumbres		
957	1081	Estofado		
958	1082	Salsa roja sin carne		
959	1083	Ensalada rusa		
960	1084	Malta lista para tomar		
961	1085	Café con leche listo para tomar		
962	1086	Mate cebado		
963	1087	Mate cocido		
964	1088	Té negro listo para tomar		
965	1089	Té de hierbas		
966	1090	Esencia de vainilla		
967	1091	Edulcorante líquido		
968	1092	Leche de avena		
969	1093	Leche de arroz		
970	1094	Leche de coco		
971	1095	Avena, almohaditas rellenas	160	
972	1096	Budín 100% de origen vegetal	179	
973	1097	Chipá 100% de origen vegetal	195	
974	1098	Churro 100% de origen vegetal	202	
975	1099	Facturas rellenas 100% de origen vegetal	208	
976	1100	Facturas simples (Medialuna salada) 100% de origen vegetal	209-a	
977	1101	Madalenas 100% de origen vegetal	278	Muffins 100% de origen vegetal
978	1102	Torta frita 100% de origen vegetal	374	
979	1103	Lechuga colorada, hoja, cruda		Lechuga morada, hoja, cruda
980	1103-a	Lechuga colorada picada cruda		Lechuga morada picada cruda
981	1104	Apio picado salteado con sal y aceite		
982	1105	Avena tostada		
983	1106	Salsa roja con carne		
984	1107	Salsa roja con soja texturizada		
985	1108	Arroz con leche		
986	1109	Compota de frutas desecadas		
987	1110	Compota de frutas frescas		
988	1111	Salsa Blanca		

N°	Food code	Food	Reference food code	Food synonyms
989	1112	Tortilla de hortalizas		
990	1113	Tarta de hortalizas con salsa blanca		
991	1114	Tarta de hortalizas con huevo		
992	1115	Tarta de soja texturizada y huevo		
993	1116	Tarta de carne picada y huevo		
994	1117	Tarta de hortalizas y salsa blanca 100% vegetal	1113	
995	1118	Tarta de pollo con salsa blanca		
996	1119	Tarta de pollo con huevo	1118	
997	1120	Tarta de jamón y queso	1118	
998	1121	Tarta de pastrón y queso	1118	
999	1122	Tarta de jamón, pastrón y queso	1118	
1000	1123	Tarta de atún y huevo	1118	
1001	1124	Tarta de queso y huevo	1118	
1002	1125	Cilantro, crudo		
1003	1125-a	Cilantro picado crudo	63-a	
1004	1126	Salsa blanca 100% vegetal	1111	
1005	1127	Pastel de papa relleno con carne		
1006	1128	Pastel de papa relleno con soja		
1007	1129	Panqueque		
1008	1130	Canelón con salsa blanca, relleno de verduras y ricota		
1009	1131	Canelón con salsa roja, relleno de carne		
1010	1132	Canelón con salsa blanca, relleno de pollo y ricota		
1011	1133	Canelón con salsa blanca, relleno verduras y tofu 100% origen vegetal		
1012	1134	Canelón con salsa roja, relleno de soja 100% origen vegetal		
1013	1135	Panqueque 100% origen vegetal	1129	
1014	1136	Salsa de tomate para pizza	81	
1015	1137	Pizza con aceituna		
1016	1138	Pizza con rodajas de tomate		
1017	1139	Pizza con huevo duro		
1018	1140	Pizza con pastrón		
1019	1141	Pizza con queso roquefort		
1020	1142	Pizza con jamón de pechuga de pollo		
1021	1143	Pizza con morrón		
1022	1144	Pizza muzzarella		
1023	1145	Pizza con jamón de cerdo		
1024	1146	Bomba de papa		
1025	1147	Pastafrola		
1026	1148	Bizcochuelo simple		
1027	1149	Agua		
1028	1150	Ribel		Torta Rusa - Riwwel Kuchen
1029	1151	Galleta de avena		
1030	1152	Milanesa a caballo de carne vacuna rebozada frita		
1031	1153	Milanesa a caballo de pollo rebozada frita	1152	
1032	1154	Milanesa a la napolitana de carne vacuna rebozada frita		
1033	1155	Milanesa a la napolitana de pollo rebozada frita	1154	
1034	1156	Puchero		
1035	1157	Locro		
1036	1158	Té de hibiscus		Té de rosa china - Agua de Jamaica - Té de Jamaica - Té de cayena
1037	1159	Ají triturado		
1038	1160	Empanada frita con relleno de carne		
1039	1161	Empanada frita con relleno de soja texturizada	1160	
1040	1162	Empanada frita con relleno de soja texturizada 100% de origen vegetal	1160	
1041	1163	Empanada frita con relleno de verdura sin huevo	1160	
1042	1164	Empanada frita con relleno de verdura con huevo	1160	
1043	1165	Empanada frita con relleno de verdura y ricota	1160	
1044	1166	Empanada frita con relleno de choclo y huevo	1160	
1045	1167	Empanada frita con relleno de choclo y salsa blanca	1160	
1046	1168	Empanada frita con relleno de verdura 100% de origen vegetal	1160	
1047	1169	Empanada frita con relleno de cebolla y queso	1160	
1048	1170	Empanada frita con relleno de queso y huevo	1160	
1049	1171	Lima		
1050	1172	Mix frutos secos, pasas de uva, almendras, nuez, castañas de cajú		
1051	1173	Pimentón en polvo		Paprika
1052	1174	Pimienta blanca		

Legend:

(purple) Foods with reference images and weights.

(blue) Liquid foods.
