# Supplementary material for: Development of an open-access food atlas with nutrient composition and recipes in Argentina: The Smart24Food Atlas
Source: PLoS One. 2026 Sep 25;21(9):e0359331. doi: 10.1371/journal.pone.0359331 (PMC13614558; doi:10.1371/journal.pone.0359331)
Supplement: Supplementary file 2 [file pone.0359331.s007.pdf]

S2 Table. Nutrient composition data sources with their identifying colors.

Source	Color
SARA2 – Argenfoods	Light Blue
SARA2 – USDA	Light Yellow
SARA2 – British Food Composition Table	Orange
SARA2 – USDA - FNDDS	Blue
SARA2 – Labels and other sources	White
SARA2 – Value assumed as "zero"	Green
SARA2 – Water calculated by difference	Grey
MAR24 – USDA	Intense Yellow
